# A multi-agent multi-modal LLM with Monte Carlo tree search for interpretable plant disease classification

**DOI:** 10.3389/fpls.2026.1937679

**Published:** 2026-09-21

**Authors:** Ridong Wu, Hua Zou, Zhijie Li, Qian Zhou, Zijiang Yi

**Affiliations:** 1 School of Computer and Information Engineering, Xi’an University of Architecture and Technology, Xi’an, China; 2 School of Computer Science, Wuhan University, Wuhan, China

**Keywords:** Monte Carlo tree search, multi-agent, multimodal information fusion, multimodal large language model, plant disease classification, self-training

## Abstract

In plant disease classification tasks, existing task specific models for plant disease classification typically achieve high accuracy by employing tailored network architectures. However, these models are unable to provide reasoning chains, which reduces interpretability and undermines trustworthiness in practical applications. In addition, although multimodal large language models (MLLMs) have made significant progress in smart agriculture, their disease classification accuracy lags behind that of task-specific models due to insufficient domain knowledge. While fine-tuning can enhance domain-specific knowledge, relying solely on unimodal data or simple multimodal information remains insufficient for adequately improving classification accuracy. Specifically, the simple multimodal information in this context refers to a single global description of the entire image produced by an MLLM. To address these issues, this paper proposes a multi-agent collaborative classification framework that integrates Monte Carlo Tree Search (MCTS) with multimodal enhancement. With an MLLM as the core, the framework first introduces an MCTSguided multi-agent collaboration mechanism that directs the agents to progressively generate region-specific descriptive textual information, which together with the image forms complex multimodal information and thereby boosts classification performance. Meanwhile, the search trajectories generated by MCTS are leveraged to construct reasoning processes for enhanced interpretability. Finally, a self-training mechanism is adopted to iteratively optimize the model, further improving overall performance. Experimental results show that the framework improves the baseline MLLM’s accuracy while providing interpretable reasoning chains, achieving gains from 0.6701 to 0.9965 on 3-class potato, 0.5340 to 0.9651 on 5-class potato, 0.5387 to 0.9467 on tomato, and 0.4705 to 0.9948 on grape.

## 1 Introduction

Plant diseases pose a major threat to global food security by reducing crop yield and product quality, with symptoms manifesting in tissues such as leaves, stems, roots, and fruits (Dai et al., 2024; Rashid et al., 2021). Foliar diseases are among the most prevalent and economically significant plant diseases, as their airborne transmission enables rapid spread, and their direct impact on photosynthetic tissue leads to substantial yield losses (Mahum et al., 2023). Early and accurate identification of these symptoms is therefore essential for timely intervention and effective field management. However, traditional disease diagnosis still relies heavily on manual inspection and expert knowledge, which is often time-consuming, costly, and difficult to scale in practical agricultural scenarios (Kaur and Sharma, 2021). Therefore, visionbased intelligent disease classification has become an important direction in agricultural research, as leaves provide the most intuitive and accessible source of disease information (Zhu et al., 2025).

Recent advances in artificial intelligence have greatly promoted plant disease detection and diagnosis (Sarkar et al., 2023). A variety of task-specific models based on visible images (Chakraborty et al., 2022) and various improved deep learning networks have been widely applied to plant disease recognition tasks. These improvements include innovations in activation functions (Chaibi et al., 2025), integration of specific modules (Ashurov et al., 2025), introduction of attention mechanisms (Reis and Turk, 2024), and design of lightweight detection architectures (Chen et al., 2026). In the area of semi-supervised learning, related methods have also shown potential under limited annotation conditions (Fan et al., 2025). These models have shown promising results in disease classification, lesion segmentation, and severity estimation. To better characterize the complexity and diversity of disease symptoms, researchers have further introduced multimodal fusion methods. A graph learning-based multimodal feature interactive fusion model was proposed (Wu et al., 2026). To address the issue of insufficient use of multimodal data in current methods, semantic embedding techniques for disease images and corresponding descriptive texts were explored (Zhou et al., 2021). Furthermore, multimodal model recognition methods integrating image, text, and label information were constructed (Cao et al., 2023), and a multimodal artificial intelligence model based on the integration of multiple modules was proposed (Zhu et al., 2025). However, despite these advances, most existing task-specific models still operate as one-step prediction models on fixed inputs and remain largely black-box in nature (Wu et al., 2025). Consequently, they provide limited insight into how a diagnosis is reached and rarely offer reasoning processes or descriptive textual evidence that are explicitly grounded in visual cues (as illustrated in **Figure 1**).

**Figure 1:**
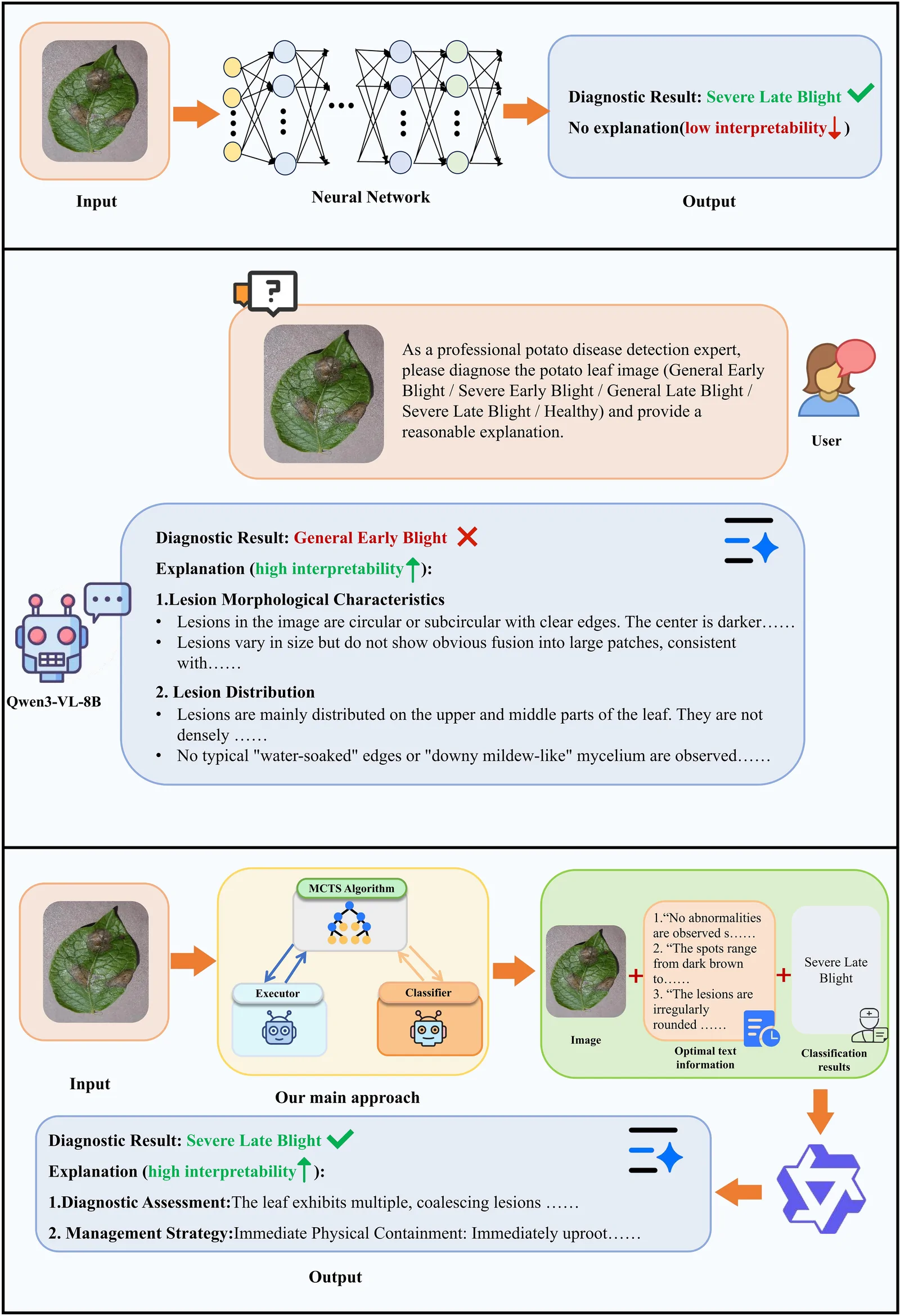
Schematic illustration of three types of models. Upper panel: task-specific models; Middle panel: MLLMs; Lower panel: our approach (red indicates misclassification or low interpretability; green indicates correct classification or high interpretability).

With the rapid advancement of MLLMs, their strength in cross-modal understanding and natural-language reasoning has opened new possibilities for improving interpretability in agricultural diagnosis, particularly by enabling the generation of human-readable diagnostic explanations (Jiang et al., 2025). Recent studies have further strengthened the visual grounding and reasoning abilities of MLLMs. For instance, pix2emb achieves unified modeling of diverse location formats, thereby enhancing spatial representation in multimodal reasoning (Zhang et al., 2023), while ROD-MLLM improves reliable classification under complex language scenarios (Yin et al., 2025). Building on these advances, MLLMs have also begun to show promise in plant disease diagnosis. LLMI-CDP, which integrates the VisualGLM architecture with Low-Rank Adaptation (LoRA) and the Q-Former framework, was proposed and demonstrated superior performance over five mainstream MLLMs on a custom dataset (Wang et al., 2025). Similarly, an MLLM-driven AI agent system for plant disease diagnosis was developed by integrating a multi-module architecture with fine-tuning on large-scale disease datasets, thereby improving multidimensional analysis and diagnostic capability (Qin et al., 2025). In addition, Agri Insight, a chatbot designed for Tamil-speaking farmers that integrates text, image, and speech inputs to support real-time disease diagnosis, was introduced (Kumar et al., 2025). However, in fine-grained plant disease diagnosis, current MLLMs still lag behind task-specific models in classification accuracy, making it difficult to simultaneously achieve superior classification performance and reliable interpretability (as illustrated in **Figure 1**). Moreover, existing domain knowledge fine-tuning methods for large models, whether based on unimodal information or multimodal information containing simple text descriptions, struggle to match the performance level of task-specific models (for details, see Section 3.5).

This limitation suggests that plant disease diagnosis should be treated not merely as a multimodal understanding problem, but also as a sequential evidence-gathering process in which informative regions need to be progressively examined before more complex and precise textual information can be made, thereby integrating more appropriate multimodal information. From this perspective, MCTS is particularly suitable because it supports sequential exploration in large decision spaces (Browne et al., 2012). Rather than making a single global judgment, MCTS can guide the model to iteratively focus on candidate regions, evaluate their diagnostic value, and refine subsequent observations. Moreover, MCTS naturally generates interpretable search trees, which aligns well with the need for transparent diagnostic reasoning. Building on this advantage, ReKG-MCTS, a knowledge graph reasoning framework incorporating MCTS, was proposed (Song et al., 2025); MCTS was applied to complex knowledge-intensive tasks, constructing a retrieval-augmented reasoning framework named RARE (Tran et al., 2025); MCTS was leveraged to address order wave scheduling problems in agricultural seed supply chains (Thangeda et al., 2025); and multiple models were further coordinated within the MCTS tree, developing a lightweight multi-LLM collaborative reasoning framework (Tang et al., 2024). Furthermore, once diagnosis is formulated as a progressive search process, the task naturally involves multiple functions, including local observation, evidence organization, and final decision-making. This makes multi-agent collaboration an effective complement, as it can decompose the overall diagnostic procedure into specialized roles, thereby improving coordination efficiency, robustness, and scalability (Chang et al., 2025). This collaborative paradigm has been widely explored in the agricultural domain, encompassing unmanned cooperative control of electric tractors (Xu et al., 2025b), a multi-agent data analysis system driven by large language models (Pan et al., 2024), multi-agent visual perception and autonomous spraying in orchards (Góral et al., 2024), and a tractor-implement multi-agent cooperative control framework (Jia et al., 2025).

Despite these advances, existing MCTS applications in plant disease diagnosis and related domains still suffer from several limitations. First, most existing works merely employ MCTS as a search tool for knowledge graph reasoning or relation verification, focusing on discovering relational paths through its exploration capability, while failing to exploit its potential for incremental acquisition and fusion of fine-grained visual evidence. Second, the search paths naturally generated by MCTS during reasoning are rich in decision trajectories, yet existing frameworks typically treat them as mere intermediate byproducts and discard them without utilization, failing to transform them into interpretable reasoning chains that can support diagnostic decisions. Third, although multi-agent systems in agriculture have made some progress in division of labor and collaboration, their decision-making processes are often disconnected from MCTS-based search planning, making it difficult to effectively transmit macro-level reasoning strategies to micro-level observational execution, leaving the utilization of multimodal information at a simple fusion level. To this end, this study is guided by the following research questions: (i) Task-specific models achieve high accuracy but lack interpretability, while the search process of MCTS naturally generates decision paths. Can we leverage this to construct traceable reasoning chains, enabling the model to achieve interpretability while maintaining high classification accuracy? (ii) MLLMs possess reasoning capabilities but underperform task-specific models in fine-grained classification. Given that MCTS can accumulate fine-grained visual evidence through stepwise exploration, can we exploit this to enhance the richness of multimodal information and thereby improve the classification performance of MLLMs? (iii) Fine-tuning based on unimodal or simple multimodal information yields limited improvement, while MCTS can dynamically select informative regions and fuse multi-source evidence according to diagnostic needs. Can we integrate this capability with multi-agent systems, enabling macro-level search strategies to effectively guide micro-level observational execution and thereby make more sufficient use of multimodal information?

Building upon the above observations and questions, this study proposes a collaborative multi-agent framework based on multimodal MCTS for plant disease classification. The framework adopts a MLLM as the perceptual foundation and introduces MCTS as the core decision-making engine to mimic the stepwise diagnostic reasoning process of human experts. Taking a plant leaf image as the initial input, under the guidance of MCTS, the execution agent attends to specific image regions in accordance with the policy produced by MCTS, transforms the observations into textual evidence, and incorporates this evidence into the multimodal information, thereby enabling iterative updates of the multimodal information. A classifier agent then produces the final diagnosis based on the progressively accumulated and enhanced multimodal information, including both visual inputs and textual decision chains. In this way, the framework achieves iterative diagnostic process that continuously refines disease understanding through sequential evidence acquisition. Specifically, unlike existing MCTS applications, our framework leverages MCTS to guide the incremental acquisition and fusion of region-specific visual descriptions, directly addressing the issue of insufficient multimodal information in plant disease diagnosis. This enables the model to progressively enhance multimodal information to improve classification accuracy. Moreover, the search paths generated by MCTS are explicitly constructed as traceable reasoning chains that support predictions with human-understandable logic, thereby offering a more transparent and reliable form of reasoning. At the system level, our framework achieves a deep coupling between MCTS-based decision planning and multi-agent collaborative execution: the executor agent translates macro-level search strategies into concrete observational actions, while the classifier agent synthesizes the accumulated evidence to make the final diagnosis. This division of labor ensures that the planning capability of MCTS is faithfully realized through specialized agent roles. Furthermore, a multi-stage self-training strategy is proposed to leverage the high-quality paths produced by MCTS in the preceding stage (Zhai et al., 2025; Xu et al., 2025a). These paths are converted into training signals and used for iterative fine-tuning, thereby enabling the model to evolve progressively over successive stages. The primary contributions of this work can be summarized as follows:

1. We propose a MCTS-guided multi-agent multimodal framework for plant disease diagnosis, which redefines the diagnostic process as a continuous evidence discovery and decision-making procedure, rather than a one-step prediction task.
2. We design a traceable reasoning mechanism in which MCTS search trajectories and multimodal interactions provide visually grounded decision paths and descriptive textual evidence, acquiring complex and precise multimodal information and improving the interpretability of diagnosis results.
3. We introduce a multi-stage self-training strategy that uses MCTS-generated multimodal decision-path data to iteratively optimize the core classifier and improve its performance.
4. The framework achieves 0.9965 on 3-class potato, 0.9651 on 5-class potato, 0.9467 on tomato, and 0.9948 on grape, while providing interpretable reasoning chains and promising cross-crop transferability.

## 2 Materials and methods

**Figure 2** illustrates the overall workflow of the proposed crop leaf disease diagnosis framework. The framework enhances multimodal information dynamically by progressively focusing on key disease features, thereby improving the classification accuracy of the MLLM. Additionally, the framework leverages the traceable reasoning trajectories generated by MCTS to make the decision process transparent and understandable, thereby providing reliable interpretability. Given an input leaf image, the system first initializes a search state consisting of the original image, an empty textual evidence chain, and an empty action history. It then enters an iterative decision loop driven by MCTS, where either observation actions or classification actions are selected at each step. Observation actions are executed by the executor agent to inspect informative regions and generate structured textual evidence, whereas classification actions are executed by the classifier agent to predict the disease category based on the current multimodal information. The search process terminates when the maximum steps is attained, and the highest-reward diagnostic path found by MCTS is returned. After diagnosis, the original image and the classification result are further provided to a MLLM to generate natural-language treatment recommendations. The final output therefore includes the predicted disease type, the corresponding reasoning trajectory, and the suggested control strategy.

**Figure 2:**
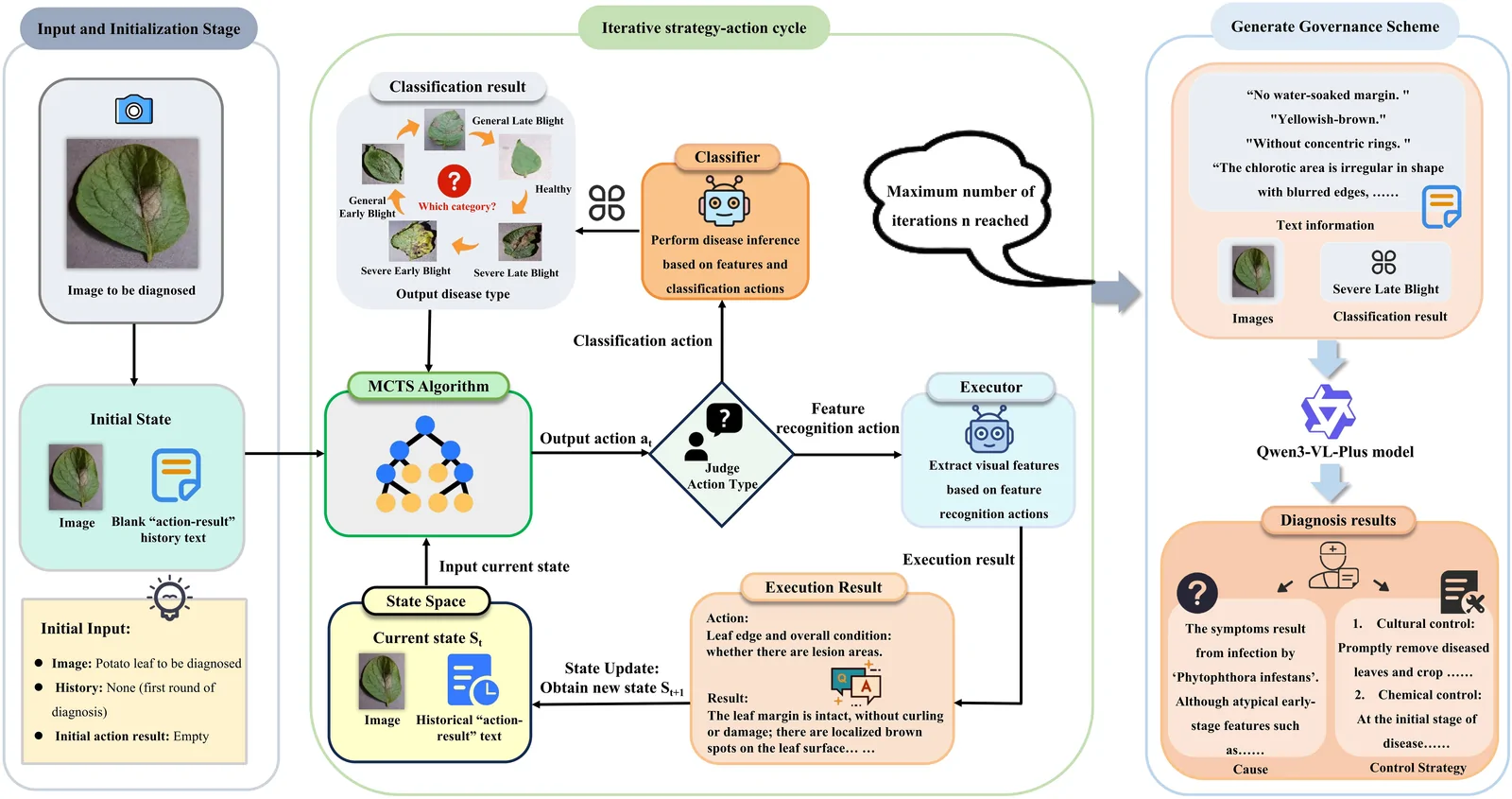
Overall architecture of our method. Given an input image, MCTS performs stepwise search and reasoning, the executor agent conducts region-specific observation, and the classifier agent produces the final diagnosis. Based on the diagnosis result, the corresponding reasoning chain, and the input image, Qwen3-VL-Plus generates a control strategy and diagnostic cause.

### 2.1 Multi-agent collaboration

Inspired by the distributed decision-making strengths of multi-agent systems, this study designs a collaborative system comprising an executor agent and a classifier agent, with MCTS serving as the central decision coordinator. The two agents have specialized and complementary functions:

1. Executor agent. As shown in **Figure 3A**, the executor agent serves as the perceptual front-end of the system. Given the current state and the action selected by MCTS, it performs observation on the input image and converts the observed visual patterns into structured textual evidence.
2. Classifier agent. As shown in **Figure 3B**, the classifier agent serves as the decision-making back-end. It takes the original image together with the accumulated textual evidence chain as input and outputs the disease category.

**Figure 3:**
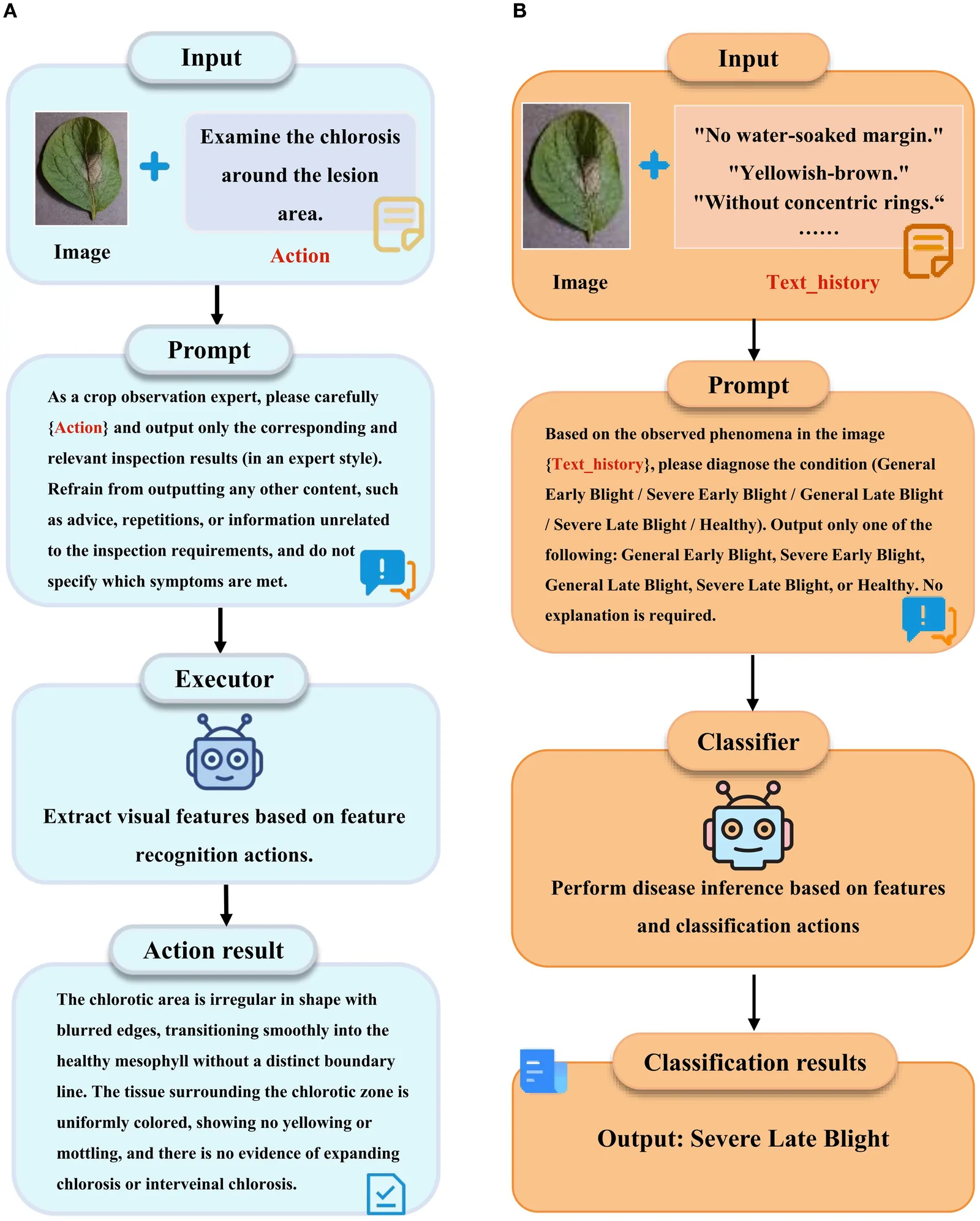
Overall workflow of the agent. **(A)** Operational details of the executor. **(B)** Operational details of the classifier.

The two agents are connected through a shared textual decision chain, which is updated throughout the search process. Starting from the initial state, MCTS selects the next action according to the current image and the existing textual evidence (see Section 2.2 for details). If an observation action is selected, the executor agent performs the corresponding visual inspection and appends the resulting textual description to the decision chain. If a classification action is selected, the classifier agent predicts the disease category based on the current multimodal information. Through their respective functions, the framework progressively refines diagnostic evidence while maintaining a traceable record of the reasoning process.

### 2.2 MCTS-based decision engine

This study formulates plant disease classification as a dynamic task and introduces MCTS as the core planner, guiding the model to perform dynamic and proactive visual reasoning, thereby progressively enhancing multimodal information. As shown in **Figure 4**, MCTS constructs a tree-structured diagnostic trajectory, where each node represents a state.

**Figure 4:**
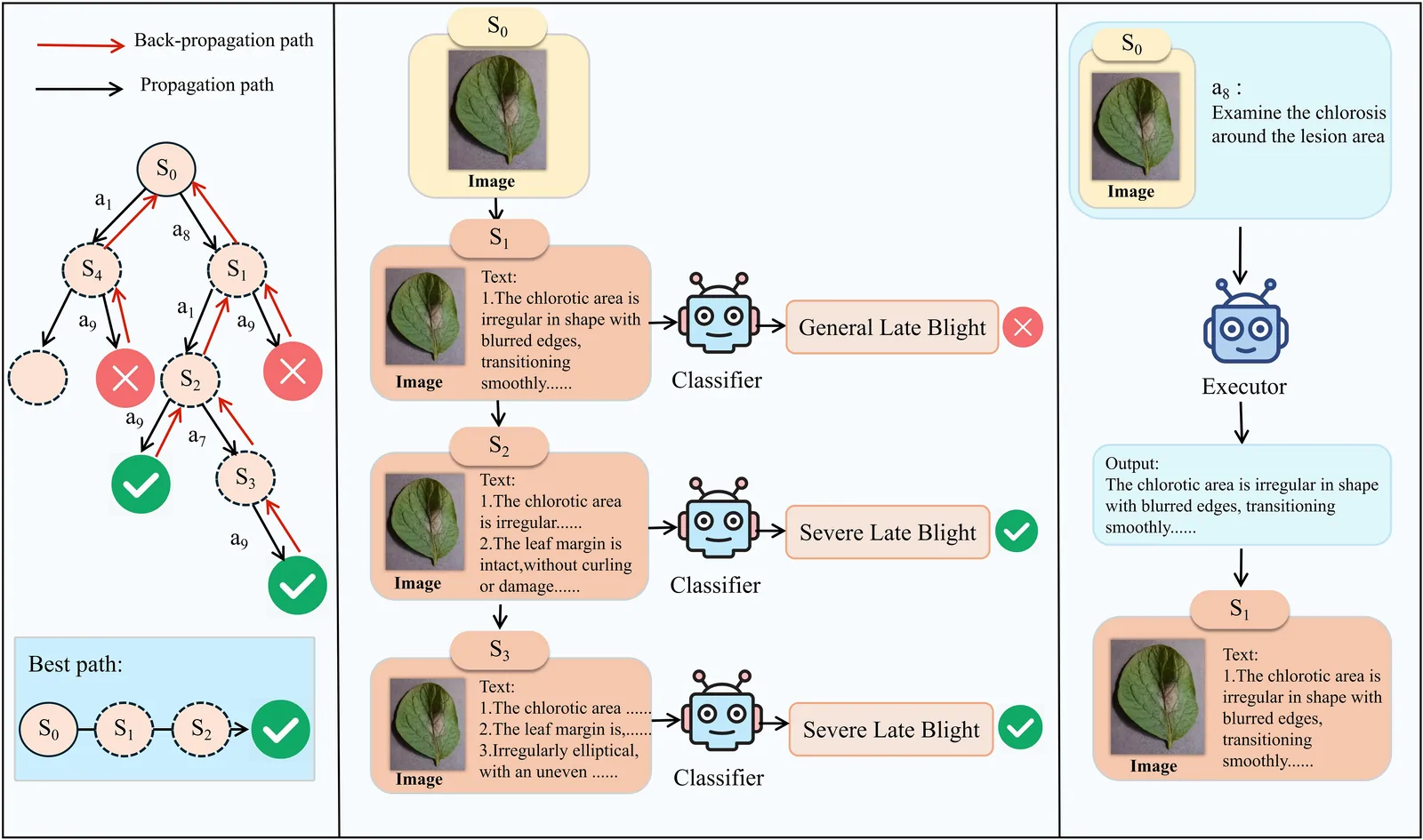
Overview of the MCTS search tree and diagnostic process. (left) Overall structure of the search tree with the best path. (middle) Detailed illustration of partial paths. (right) State transition process from node *S*_0_ to *S*_1_. *S* denotes a state or node in the search tree.

In this framework, the core elements of MCTS are defined as follows:

1. Action Space: As shown in **Table 1**, the value of “Action” indicates the key focus of observation. These actions have been defined, filtered, and optimized by experts. Furthermore, “A” in the **Table 1** and “a” mentioned in the following content both represent the action ID. The detailed mechanisms for action selection and execution will be introduced in detail later. It should be noted that the action space shown in **Table 1** is crop-specific and was developed exclusively for potato disease diagnosis. For tomato and grape datasets, experts customize the actual action sets according to the pathological characteristics of each crop;
2. State Space: As shown in **Table 2**, the state space consists of two components: the visual state and the textual state. The visual state is the input query image, which remains fixed throughout the entire diagnostic process. The textual state is a string that stores the observation text generated by executing the action selected via MCTS. Unlike the visual state, the textual state is dynamically updated as new observations are continuously appended during the iterative reasoning process;
3. Reward Function: As shown in **Table 3**, the reward function consists of two phases. During training, the reward for potato has five components: +50 for correct classification, -30 for wrong classification, -15 for grading error, -5 per action step, and +0.5 per valid observation. Specifically, the reward values are designed to prioritize final classification accuracy, with a larger positive reward for correct predictions (+50) to strongly incentivize accurate outcomes. Penalties for incorrect predictions (-30) and severity errors (-15) are set at smaller magnitudes, reflecting that misclassifying disease severity is less severe than a completely wrong prediction. A per-step penalty (-5) encourages the model to learn concise reasoning paths by penalizing unnecessarily long sequences, while a per-observation reward (+0.5) provides mild positive feedback to facilitate effective information acquisition. During testing, all three crops (potato, tomato, and grape) share the same confidence-based reward, which is computed via average log-likelihood, softmax probability, and the confidence gap (Geng et al., 2024). Specifically, during testing, the classifier agent assigns a reward to the current path based on its confidence in the predicted category.

**Table 1:** Detailed definition of action space in potato.

ID	Action
A1	Leaf edge and overall condition: whether there are lesion areas
A2	Whether the lesion area exhibits concentric rings
A3	Whether the lesion edge appears water-soaked
A4	The proportion of the lesion area
A5	The specific location of the lesion area
A6	The specific color of the lesion area
A7	The specific shape of the lesion area
A8	Chlorosis around the lesion area
A9	Output the final classification result

**Table 2:** Detailed description of the state space.

State component	Data type	Description
Visual State	Image	The query image to be diagnosed (remains fixed throughout the process)
Textual State	String	Observation text generated by executing the action selected via MCTS

**Table 3:** Detailed definition of reward function in potato.

Stage	Reward component	Value or formula	Description
Train	Correct classification	+50	Exactly matches true label
	Wrong classification	-30	Exactly mismatches the true label
	Grading error	-15	Only disease type matches
	Per-step penalty	-5	Per-step penalty for concise paths
	Valid observation	+0.5	Small reward for each action
Test	Classification Confidence	$\text{avg\_ll}\left(c\right)\;=\;\frac{1}{T_{c}}\sum_{i=1}^{T_{c}}\log\;P\left({\text{token}}_{i}\;|\;{\text{prompt, token}}_{1:i-1}\right)$	Average Log-Likelihood
		$P\left(c\right)=\frac{\text{exp}\;(\text{avg}\_\text{ll}\left(c\right)-\max_{c^{\prime }}\text{avg}\_\text{ll}\left(c^{\prime }\right))}{\sum_{c^{\prime }}\exp\;(\text{avg}\_\text{ll}\left(c^{\prime }\right)-\max_{c^{\prime }}\text{avg}\_\text{ll}\left(c^{\prime }\right))}$	Softmax Probability
		$100\times \left(\max_{c}\;P\left(c\right)-\text{second }{\text{max}}_{c}P\left(c\right)\right)$	Confidence Gap Reward

*T_c_* is the token length of class *c*’s label; *P*(token*_i_* | prompt,token_1:_*_i_*_−1_) denotes the model’s predicted probability of the *i*-th token; avg_ll(*c*) is the average log-likelihood of generating the full label of class *c*; *P*(*c*) is the softmax probability over classes after normalization; max*_c_ P*(*c*) and secondmax*_c_ P*(*c*) represent the highest and second-highest class probabilities.

Selection. The first phase of MCTS is the selection phase, where the search starts from the root node (*S*_0_ in **Figure 4**). In each iteration, the next node is selected according to the Upper Confidence Bounds Applied to Trees (UCT) value of its child nodes until a node that is incompletely expanded is reached.

For a child node *S*′ of the current node *S*, the UCT value is computed as follows:

(1) $$\text{UCT}\left(S^{\prime }\right)=\frac{Q\left(S^{\prime }\right)}{N\left(S^{\prime }\right)}+C\cdot \sqrt{\frac{\text{ln}\;N\left(S\right)}{N\left(S^{\prime }\right)}}$$

where *Q*(*S*′) is the cumulative reward (please see Equation 1). More precisely, each time the search process visits state *S*′ during a simulation, the final reward obtained from that complete diagnostic trajectory is accumulated into *Q*(*S*′) (the simulation process and backpropagation process are described in detail later). *N*(*S*′) denotes the visit count of the child node, and *N*(*S*) denotes the visit count of the current (parent) node. Following the common practice (Silver et al., 2017), we choose *C* = 1.4 by default. If *N*(*S*′) = 0, then UCT(*S*′) = ∞. The selection rule for the current node *S* is as follows (please see Equation 2):

(2) $$S^{*}=\underset{S^{\prime }\in \text{Children}\left(S\right)}{\arg\max}\text{UCT}\left(S^{\prime }\right)$$

Starting from the root node *S*_0_, the above rule is applied recursively to descend layer by layer until an incompletely expanded node is reached, which will be used for subsequent expansion. If only the root node is present, the selection step directly returns the root node.

Expansion. The second phase of MCTS is the expansion phase, which expands the node output from the selection phase. In general, starting from a selected node, an untried action is selected and executed to generate a new child node. As shown in **Figure 4**, starting from the root node *S*_0_, action *a*_8_ is selected and executed, resulting in a new node *S*_1_.

Let the selected node output from the selection phase be *S*_cur_, whose set of untried actions is $A_{\text{untried}}\left(S_{\text{cur}}\right)\neq \emptyset$. An action is selected from this set (please see Equation 3):

(3) $$a_{\text{selected}}\in A_{\text{untried}}\left(S_{\text{cur}}\right)$$

When executing the action, the corresponding function is called according to the action type to update the state and generate a new child node:

(4) $$S_{\text{new}}=\{\begin{array}{ll} \text{Executor}\left(S_{\text{cur}},a_{\text{selected}}\right), & \text{if }a_{\text{selected}}\text{ is observation}\\ \text{Classifier}\left(S_{\text{cur}},a_{\text{selected}}\right), & \text{if }a_{\text{selected}}\text{ is classification} \end{array}$$

Here, Executor(·,·) is the executor function that executes observation actions and returns a new state; Classifier(·,·) is the classifier function that executes classification actions and returns a new state (please see Equation 4). The newly expanded node *S*_new_, which becomes a child node of *S*_cur_, proceeds to the next phase.

Simulation. The third phase of MCTS is the simulation phase, which is triggered only when the expansion phase executes an observation action. Starting from the newly expanded node *S*_new_, a random simulation of subsequent actions is performed until a classification action or a terminal state is reached, and the classifier evaluates the result to obtain a reward. This phase only performs path rollouts and does not generate new nodes.

Let *S*_new_ be the node expanded from an observation action. A random simulation is performed from this node:

(5) $$S_{T}=\text{Simulate}\left(S_{\text{new}}\right)$$

where Simulate(·) is the random simulation function (please see Equation 5). Starting from the newly expanded node *S*_new_, it repeatedly and uniformly selects an action at random from $A_{\text{untried}}\left(S_{\text{new}}\right)$ and executes it. As actions are selected, $A_{\text{untried}}\left(S_{\text{new}}\right)$ is updated synchronously. Notably, during this simulation phase, no new nodes are expanded or added to the search tree. It stops when a classification action or a termination condition is reached, and returns the terminal state *S_T_*. Upon reaching the terminal state, the classifier is invoked for evaluation. The reward is computed based on the classification result and the simulation path:

(6) $$\hat{y}=\text{Classifier}\left(S_{T}\right),\;r=ℛ\left(\text{Path}\left(S_{\text{new}}\to S_{T}\right),\hat{y}\right)$$

where Classifier(·) is the classifier function that outputs the prediction (see Equation 6) $\hat{y}$, $\text{Path}\left(S_{\text{new}}\to S_{T}\right)$ denotes the simulation path from *S*_new_ to *S_T_* and ℜ(·,·) is the reward function that computes a scalar reward value *r* based on the simulation path and the prediction, following the reward design rules defined in **Table 3**.

The reward value *r* will be used in the subsequent backpropagation phase. If the expansion phase executes a classification action, no simulation is performed. In the **Figure 4**, *a*_9_ is a classification action, while the remaining actions are observation actions.

Backpropagation. The fourth phase of MCTS is the backpropagation phase (feedback). The reward value *r* obtained from the simulation phase is propagated upward along the visited path, updating only the statistics of the visited nodes.

Let the visited path be (*S*_0_*,S*_1_*,…,S*_new_). For each node *S* on the path, the following update is performed (see Equation 7):

(7) Q(S)←Q(S)+r, N(S)←N(S)+1

where *Q*(*S*) represents the cumulative reward of node *S*, and *N*(*S*) denotes its visit count.

After backpropagation is completed, the current MCTS iteration ends. The algorithm goes back to the selection phase and begins the next iteration until a preset number of iterations or a termination condition is reached.

Optimal Path Selection. The fifth phase of MCTS is optimal path selection. After multiple MCTS iterations, the algorithm outputs the optimal path from the search tree. The selection criterion for the optimal path is: under the premise of ensuring correct classification, the path with the shortest length is preferred. As shown by the “Best path” in **Figure 4**, this path achieves correct disease classification with the fewest observation actions.

Let P be the set of all paths from the root node *S*_0_ to classification action nodes in the search tree T. For any path p∈P, let $\hat{y}\left(p\right)$ denote its classification result, *y* denote the true label, |p| denote the path length, and $\text{Conf}(\hat{y}\left(p\right)|p)$ denote the model’s predictive confidence for the classification result $\hat{y}\left(p\right)$ conditioned on the reasoning path *p*. The optimal path p^*^ is defined as follows (Equation 8):

(8) $$p^{*}=\{\begin{array}{ll} \underset{p\in P}{\arg\min}\;\left|p\right|\;s.\text{t}.\;\hat{y}\left(p\right)=y, & \left(\text{Training phase}\right)\\ \underset{p\in P}{\arg\max}\;\mathrm{Conf}(\hat{y}\left(p\right)|p), & \left(\text{Testing phase}\right) \end{array}$$

The action sequence corresponding to the final output optimal path *p*^∗^ is the optimal diagnostic strategy recommended by the model. The node sequence and evidence text along this path collectively form an interpretable reasoning chain.

In our implementation, the maximum number of MCTS iterations is set to n=1500 across all datasets, the maximum tree depth is set to be consistent with the number of actions in the action space, and each node is expanded at most once per iteration with one simulation per expansion.

This process enables the model to progressively focus on disease-relevant features and refine diagnostic evidence through stepwise search. At the same time, the resulting action sequence and textual evidence chain provide an explicit reasoning path and more complex and precise multimodal information, making the diagnostic process more transparent and interpretable.

### 2.3 Self-training

As shown in **Figure 5**, to further improve the classifier agent, we introduce a multi-stage self-training strategy based on MCTS-generated decision paths.

**Figure 5:**
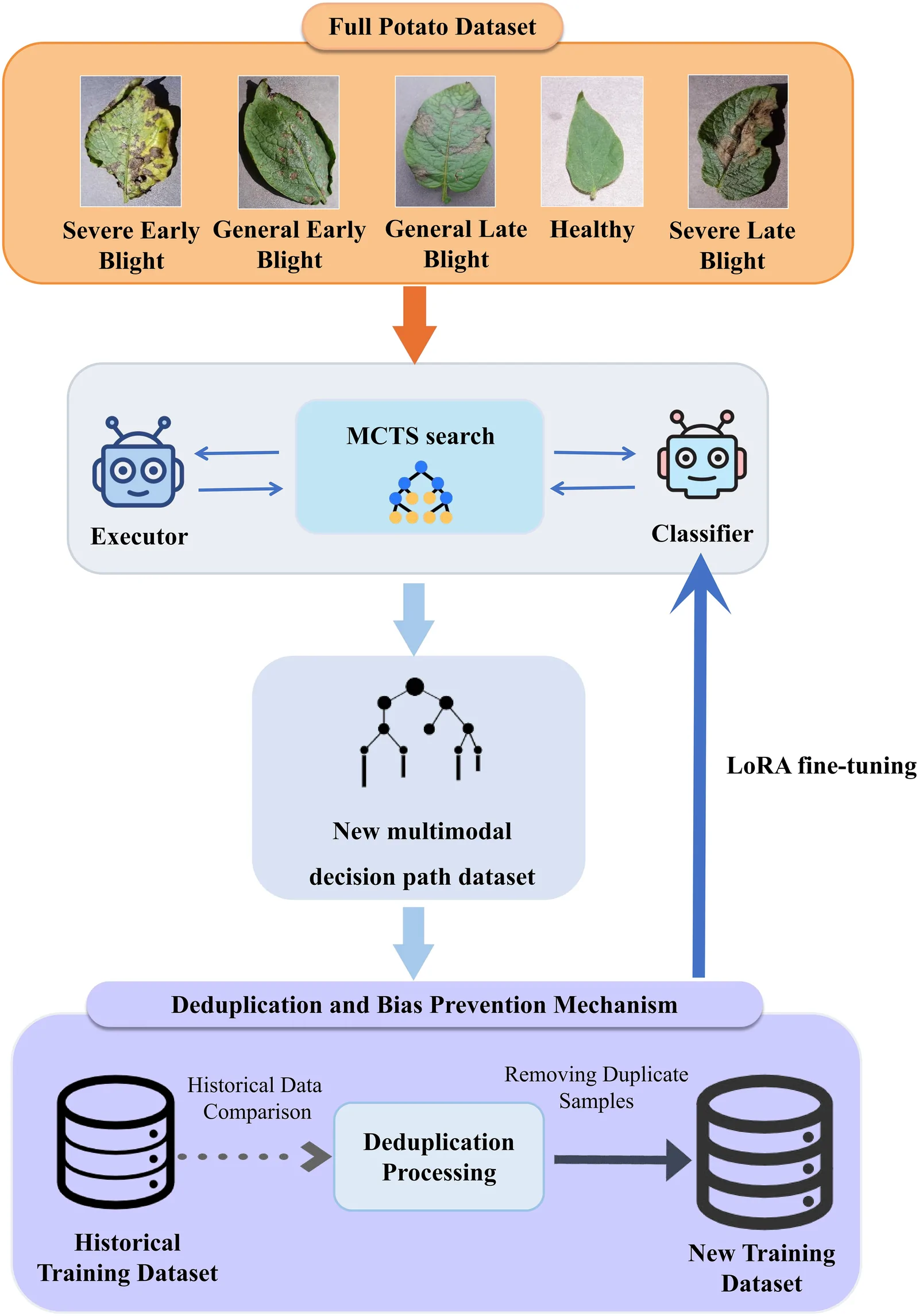
Overview of a single iteration of the MCTS-guided self-training pipeline. Given the training dataset collected via MCTS from the previous stage’s model, the data is first deduplicated. The resulting dataset is then used to perform LoRA fine-tuning on the previous round’s model, producing the updated model for the next iteration.

Specifically, given an initial classifier, MCTS is first applied to each training image to generate a sequence of observation actions along with the corresponding textual evidence chain. Each resulting sample consists of the input image, the action sequence, the accumulated evidence, and the final disease label, forming a decision-path dataset.

This dataset is then used to fine-tune the classifier agent, enabling it to learn how to perform diagnosis based on structured evidence rather than a single global representation.

Importantly, the updated classifier is further used to guide the next stage of MCTS search. Since the classifier is responsible for evaluating terminal states and providing reward signals, a stronger classifier leads to more accurate reward estimation, which in turn enables MCTS to discover higher-quality observation paths.

This process is repeated iteratively in a search–generate–fine-tune loop, allowing both the quality of decision paths and the performance of the classifier to improve progressively over multiple stages.

To avoid redundancy, newly generated samples are deduplicated against previously collected data by removing identical image–path pairs before fine-tuning.

### 2.4 Treatment recommendation generation

After obtaining the final disease diagnosis, we further generate treatment recommendations using Qwen (Bai et al., 2025). Specifically, the input image, predicted disease category, and the corresponding textual evidence chain are combined into a structured prompt, as illustrated in **Figure 6**.

**Figure 6:**
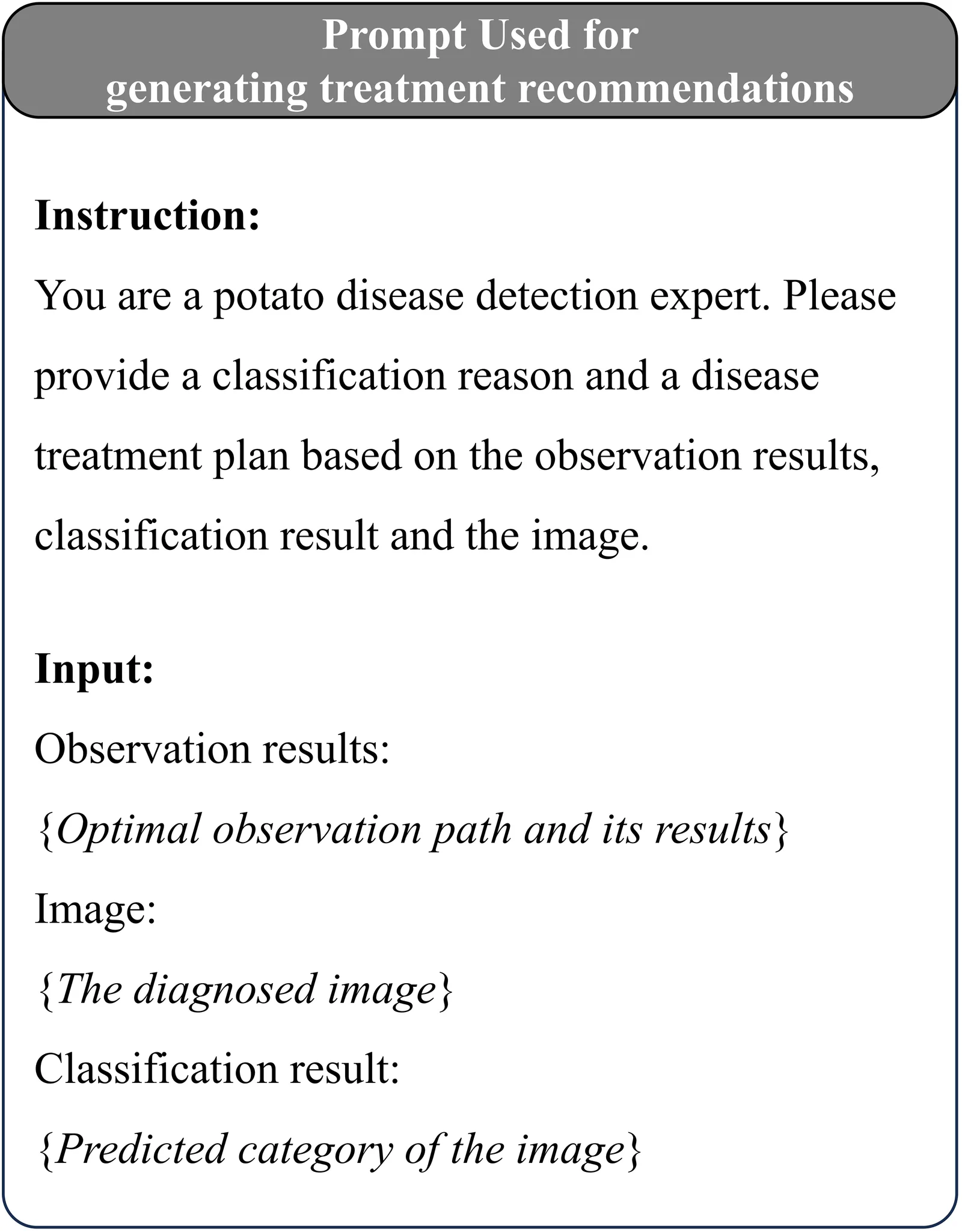
Prompt used for treatment recommendation.

Based on this input, the model produces a natural-language response that includes: (1) the identified disease, (2) the supporting diagnostic evidence, and (3) recommended control strategies.

This module transforms the diagnostic output into an actionable and interpretable decision support report, enabling the framework to assist not only in disease recognition but also in practical crop management, further improving interpretability by providing clear, traceable reasoning behind each recommendation.

## 3 Results

### 3.1 Datasets and evaluation metrics

To evaluate the proposed framework on plant leaf disease diagnosis tasks across multiple species, three datasets were used: a potato leaf disease dataset (Zhu et al., 2025) and tomato and grape leaf disease datasets from PlantVillage (Mohanty et al., 2016). The potato dataset contains five categories, namely Healthy with 500 samples, S.E.B with 510 samples, G.E.B with 203 samples, G.L.B with 251 samples, and S.L.B with 446 samples. A three-class version of this dataset was also used by merging the two Early Blight categories into a single Early Blight class and the two Late Blight categories into a single Late Blight class, resulting in Early Blight, Late Blight and Healthy. The tomato dataset used in our study includes five categories, namely L.B, B.S, E.B, Y.L.C.V, and Healthy, with 500 samples in each category.

The grape dataset contains four categories, namely B.R, L.B, Healthy, and Esca, with 500, 500, 423, and 500 samples, respectively. Detailed statistics for all datasets are provided in **Table 4**. Each dataset was partitioned into training, validation, and test splits at an 8:1:1 ratio, with this division applied consistently across all categories.

**Table 4:** Dataset distribution for training, validation, and testing.

Disease	Category	Train	Validation	Test	Total
Solanaceous crops					
Potato	G.E.B	162	21	20	203
	G.L.B	201	25	25	251
	Healthy	400	50	50	500
	S.E.B	408	51	51	510
	S.L.B	357	44	45	446
Tomato	B.S	400	50	50	500
	E.B	400	50	50	500
	Healthy	400	50	50	500
	L.B	400	50	50	500
	Y.L.C.V	400	50	50	500
Non-solanaceous crop					
Grape	B.R	400	50	50	500
	L.B	400	50	50	500
	Healthy	339	42	42	423
	Esca	400	50	50	500

S.E.B (Severe Early Blight); S.L.B (Severe Late Blight); G.E.B (General Early Blight); G.L.B (General Late Blight); E.B (Early Blight); B.S (Bacterial Spot); L.B (Late Blight for Tomato, Leaf Blight for Grape); B.R (Black rot); Y.L.C.V (Yellow Leaf Curl Virus); Esca (Black Measles).

For objective performance assessment of our proposed framework, a series of commonly used classification metrics were adopted, including precision, accuracy (ACC), recall, geometric mean (GM), F1-score (F1), and Matthews correlation coefficient (MCC), with ACC serving as the principal evaluation metric. These metrics are defined as follows (Equations 9–14):

(9) $$ACC=\frac{TP+TN}{TP+TN+FP+FN}$$

(10) $$Precision=\frac{TP}{TP+FP}$$

(11) $$Recall=\frac{TP}{TP+FN}$$

(12) $$F1\;\text{Score}=\frac{2\times TP}{2\times TP+FP+FN}$$

(13) $$GM=\sqrt{\frac{TP}{TP+FN}\times \frac{TN}{TN+FP}}$$

(14) $$MCC=\frac{TP\times TN-FP\times FN}{\sqrt{\left(TP+FP\right)\left(TP+FN\right)\left(TN+FP\right)\left(TN+FN\right)}}$$

where TP (True Positive) and TN (True Negative) represent the correctly classified positive and negative samples, respectively, while FP (False Positive) and FN (False Negative) indicate the corresponding misclassifications.

### 3.2 Experimental setup and details

All experiments were conducted on a platform equipped with eight NVIDIA RTX 3090 GPUs. The open-source Qwen3-VL-8B (Bai et al., 2025) was used as the baseline vision-language model, while Qwen3VL-Plus was employed to generate disease management recommendations. The main hyperparameter settings for model fine-tuning are summarized in **Table 5**. Additionally, the detailed sample sizes for different self-training stages across various crop datasets are reported in **Table 6**.

**Table 5:** Key hyperparameter settings for fine-tuning.

Hyperparameter	Stage 1	Stage 2	Later stages
Batch size (per GPU)	16	16	16
Learning rate	2e-5	1e-5	1e-5
LoRA alpha	32	32	16
LoRA rank	8	8	4
Epochs	5	10	3

**Table 6:** Statistics of training samples by crop and stage.

Category	Stage 1	Stage 2	Stage 3	Stage 4
Potato	2021	780	1229	634
Tomato	2341	2259	526	1872
Grape	853	1606	1726	1713

### 3.3 Performance comparison with other methods

To thoroughly evaluate the effectiveness of our method in potato disease classification tasks, we systematically reviewed recent studies in the field of potato leaf disease recognition and summarized the performance data of various works in **Table 7**. This table covers representative achievements under different datasets, diverse model architectures, and both 3-class and 5-class task settings.

**Table 7:** Performance of our method and other approaches in potato disease classification. Our results are reported as mean ± standard deviation over 3-fold cross-validation.

Method	Model	Class	ACC	F1-score	Precision	Recall
(Pandiri et al., 2022)	ODNet	3	0.9667	0.9660	0.9666	0.9663
(Chakraborty et al., 2022)	VGG-16(fine tuned)	3	0.9789	0.9617	0.9721	0.9613
(Shaheed et al., 2023)	EfficientRMT-Net	3	0.9912	–	–	–
(Al-Adhaileh et al., 2023)	CNN (customised)	3	0.9900	0.9900	0.9900	0.9900
(Arshad et al., 2023)	PLDPNet	3	0.9866	0.9633	0.9600	0.9633
(Reis and Turk, 2024)	MDSCIRNet feature & SVM	3	0.9933	0.9934	0.9933	0.9935
Our approach	Qwen3-VL-8B	**3**	**0.9965** (**± 0.0030)**	**0.9969** (**± 0.0027)**	**0.9969** (**± 0.0027)**	**0.9969** (**± 0.0027)**
(Sholihati et al., 2020)	VGG-16	5	0.9100	0.8880	0.8840	0.8920
(Mahum et al., 2023)	DenseNet (customized)	5	0.9720	**0.9867**	**0.9809**	**0.9928**
(Zhu et al., 2025)	CT-CNN & MSC-ResViT & TextCNN	5	**0.9843**	0.9810	0.9786	0.9836
Our approach	Qwen3-VL-8B	5	0.9651 (± 0.0030)	0.9583 (± 0.0045)	0.9736 (± 0.0021)	0.9475 (± 0.0058)

Best results are in bold.

In the 3-class classification task, our method achieves an accuracy of 0.9965 on the Potato Leaf Disease Dataset, outperforming all compared methods, including 0.9933 (Reis and Turk, 2024), 0.9912 (Shaheed et al., 2023), and 0.9900 (Al-Adhaileh et al., 2023). It is worth noting that in the more challenging 5-class scenario, our method based on Qwen3-VL-8B achieves an accuracy of 0.9651 while enhancing model interpretability. This result is slightly lower than the 0.9843 reported by task-specific models (Zhu et al., 2025). These results indicate that our method attains leading accuracy in the simpler 3-class task, while in the more challenging 5-class task, it attains high performance on both accuracy and interpretability. Additionally, we compared the output of our method with that of the MLLM, with a specific example shown in **Figure 7**.

**Figure 7:**
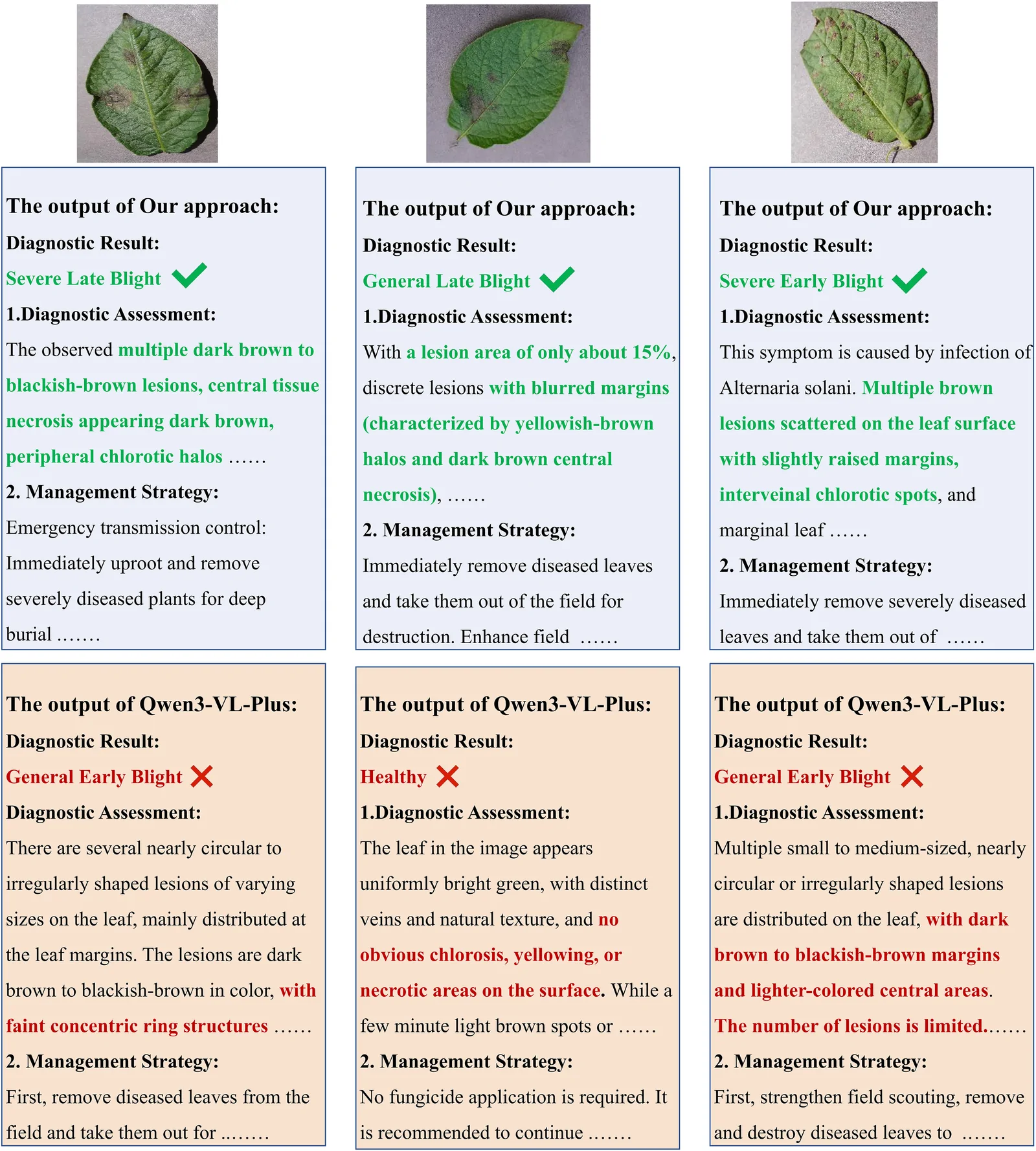
Example of output comparison between our method and MLLM, where the color coding (red/green) was performed by experts (red indicates misclassification or misinterpretation; green indicates correct classification or correct interpretation).

Additionally, to ensure the reliability of our experimental results, we performed all experiments using 3-fold cross-validation and report the mean accuracy along with the standard deviation. As shown in **Table 7**, our method achieves a mean accuracy of 0.9965 (± 0.0030) on the 3-class potato dataset and 0.9651 (± 0.0030) on the 5-class potato dataset. The relatively small standard deviations across all metrics, including an F1-score of ±0.0027 for the 3-class task and ±0.0045 for the 5-class task, indicate that our method maintains stable performance across different data folds, demonstrating its robustness and reliability.

### 3.4 Ablation study

To evaluate the contribution of each component in our framework, we conduct ablation experiments on MCTS guidance and self-training strategies (**Table 8**), comparing the performance with/without MCTS guidance under different self-training iterations (ST-R1 to ST-R4). The baseline is the original MLLM. It should be noted that the executor and classifier are not independent modules; removing either module would disable MCTS. Therefore, running without MCTS is equivalent to single-agent operation.

**Table 8:** Ablation study on MCTS guidance and multi-stage self-training strategies.

MCTS	FT Strategy	Potato		Tomato	Grape
		5-Class ACC	3-Class ACC	ACC	ACC
✗	✗	0.5340 (± 0.0091)	0.6701 (± 0.0091)	0.5387 (± 0.0092)	0.4705 (± 0.0030)
✓	✗	0.9040 (± 0.0061)	0.9319 (± 0.0060)	0.5787 (± 0.0023)	0.4740 (± 0.0052)
✗	ST-R1	0.7591 (± 0.0052)	0.8900 (± 0.0028)	0.7360 (± 0.0040)	0.4827 (± 0.0060)
✓	ST-R1	0.9058 (± 0.0053)	0.9791 (± 0.0061)	0.8053 (± 0.0101)	0.5312 (± 0.0138)
✗	ST-R2	0.8708 (± 0.0030)	0.9756 (± 0.0031)	0.8787 (± 0.0023)	0.9843 (± 0.0030)
✓	ST-R2	0.9602 (± 0.0036)	0.9965 (± 0.0030)	0.9373 (± 0.0023)	0.9861 (± 0.0030)
✗	ST-R3	0.9494 (± 0.0031)	0.9843 (± 0.0054)	0.8947 (± 0.0046)	0.9896 (0.0025)
✓	ST-R3	0.9651 (± 0.0030)	0.9895 (± 0.0042)	0.9227 (± 0.0140)	0.9948 (± 0.0031)
✗	ST-R4	0.9284 (± 0.0030)	0.9756 (± 0.0031)	0.9333 (0.0061)	0.9583 (± 0.0060)
✓	ST-R4	0.9459 (± 0.0030)	0.9773 (± 0.0031)	0.9467 (± 0.0023)	0.9618 (± 0.0030)

ST-Rn is a self-training classification agent (Qwen3-VL-8B) at stage n. Our results are reported as mean ± standard deviation over 3-fold cross-validation.

The results in **Table 8** demonstrate that applying MCTS brings performance improvements across all three datasets, validating the effectiveness of the MCTS-guided stepwise evidence acquisition mechanism (see Section 3.6 for more detailed analysis). Furthermore, self-training without MCTS also progressively improves performance as iteration rounds increase, indicating that the self-training mechanism alone can enhance the model’s diagnostic capability (see Section 3.9 for more detailed analysis). Most importantly, the combination of MCTS guidance and self-training consistently achieves the best results across all datasets, reaching 0.9651, 0.9965, 0.9467, and 0.9948 on 5-class potato, 3-class potato, tomato, and grape, respectively. These results collectively demonstrate that MCTS guidance and iterative self-training are complementary, jointly contributing to the superior performance of our framework. Detailed classification results for tomato and grape are provided in Section 3.7 and Section 3.8, respectively.

In addition, as presented in **Table 8**, we report the mean accuracy and standard deviation for each configuration based on 3-fold cross-validation. The standard deviations across all settings remain consistently small, generally below 0.01, indicating that the observed performance variations are limited and the results are statistically stable. For instance, on the 5-class potato dataset, the full configuration (MCTS + ST-R4) achieves a mean accuracy of 0.9651 with a standard deviation of ±0.0030, while the baseline without MCTS and self-training yields 0.5340 (± 0.0091). Similar patterns are observed on tomato (± 0.0140 at the highest performance) and grape (± 0.0031), further confirming the robustness of our experimental results. Additionally, the results in the following experiments are from a single fold of the 3-fold cross-validation.

### 3.5 Performance of different fine-tuning strategies and MCTS in potato disease classification

The experimental results for potato disease classification using different fine-tuning strategies and MCTS are presented in **Tables 9**–**11**. **Table 9** summarizes the accuracy for both 5-class and 3-class classification tasks across different methods, while **Tables 10**, **11** provides a detailed breakdown of the 5-class classification performance, including confusion matrices and evaluation metrics such as MCC, F1-score, and GM.

**Table 9:** Performance comparison of different fine-tuning strategies and MCTS on the potato dataset using Qwen3-VL-8B as the base model.

MCTS	FT Strategy	5-Class ACC	3-Class ACC
✗	✗	0.5235	0.6596
✓	✗	0.9110	0.9319
✗	Visual FT	0.5968	0.6335
✓	Visual FT	0.6073	0.6597
✗	Multimodal FT	0.5916	0.6544
✓	Multimodal FT	0.6283	0.6911
✗	ST-R1	0.7643	0.8900
✓	ST-R1	0.9058	0.9791
✗	ST-R2	0.8691	0.9738
✓	ST-R2	0.9581	**0.9948**
✗	ST-R3	0.9476	0.9843
✓	ST-R3	**0.9686**	0.9895
✗	ST-R4	0.9319	0.9791
✓	ST-R4	0.9424	0.9738

ST-Rn is a self-training classification agent (Qwen3-VL-8B) at stage n. Visual FT and Multimodal FT are fine-tuning methods based on Qwen3-VL-8B. Best results are in bold.

**Table 10:** Detailed performance comparison of different fine-tuning strategies and MCTS on 5-class potato dataset using Qwen3-VL-8B as the base model. (Part 1/2).

MCTS	FT strategy	Confusion matrices						Performance evaluation		
		Actual↓/Predict→	S.E.B	G.E.B	Healthy	G.L.B	S.L.B	MCC	F1	GM
✗	✗	S.E.B	26	25	0	0	0	0.3500	0.5200	0.6527
		G.E.B	0	20	0	0	0	0.3819	0.3810	0.7873
		Healthy	0	0	50	0	0	0.9736	0.9804	0.9929
		G.L.B	0	19	2	4	0	0.3253	0.2667	0.3988
		S.L.B	23	21	0	1	0	0.0000	0.0000	0.0000
✓	✗	S.E.B	46	3	0	0	2	0.9191	0.9388	0.9463
		G.E.B	0	20	0	0	0	0.8615	0.8696	0.9823
		Healthy	0	0	50	0	0	1.0000	1.0000	1.0000
		G.L.B	0	2	0	16	7	0.7509	0.7619	0.7976
		S.L.B	1	1	0	1	42	0.8362	0.8750	0.9358
✗	Visual FT	S.E.B	45	6	0	0	0	0.5173	0.6522	0.7859
		G.E.B	1	19	0	0	0	0.5207	0.5278	0.8756
		Healthy	0	0	50	0	0	0.9736	0.9804	0.9929
		G.L.B	1	22	2	0	0	0.0000	0.0000	0.0000
		S.L.B	40	5	0	0	0	0.0000	0.0000	0.0000
✓	Visual FT	S.E.B	45	6	0	0	0	0.5173	0.6522	0.7859
		G.E.B	1	19	0	0	0	0.5280	0.5352	0.8788
		Healthy	0	0	50	0	0	0.9866	0.9901	0.9964
		G.L.B	1	22	1	1	0	0.1869	0.0769	0.2000
		S.L.B	40	4	0	0	1	0.1307	0.0435	0.1491
✗	Multimodal FT	S.E.B	39	12	0	0	0	0.4751	0.6290	0.7609
		G.E.B	0	20	0	0	0	0.4875	0.4819	0.8652
		Healthy	0	0	50	0	0	0.9866	0.9901	0.9964
		G.L.B	0	20	1	4	0	0.3769	0.2759	0.4000
		S.L.B	34	11	0	0	0	0.0000	0.0000	0.0000
✓	Multimodal FT	S.E.B	40	10	0	0	1	0.4917	0.6400	0.7706
		G.E.B	0	20	0	0	0	0.5310	0.5263	0.8885
		Healthy	0	0	50	0	0	1.0000	1.0000	1.0000
		G.L.B	0	21	0	4	0	0.3769	0.2759	0.4000
		S.L.B	34	5	0	0	6	0.2857	0.2308	0.3639
✗	ST-R1	S.E.B	45	5	0	0	1	0.7811	0.8411	0.9017
		G.E.B	1	19	0	0	0	0.7031	0.7170	0.9339
		Healthy	0	0	50	0	0	0.9866	0.9901	0.9964
		G.L.B	0	8	1	16	0	0.4687	0.5424	0.7554
		S.L.B	10	1	0	18	16	0.5197	0.5161	0.5942
✓	ST-R1	S.E.B	47	2	0	1	1	0.9191	0.9400	0.9531
		G.E.B	1	19	0	0	0	0.8943	0.9048	0.9661
		Healthy	0	0	50	0	0	1.0000	1.0000	1.0000
		G.L.B	0	1	0	24	0	0.7656	0.7869	0.9437
		S.L.B	1	0	0	11	33	0.8060	0.8354	0.8534
✗	ST-R2	S.E.B	48	2	0	1	0	0.8591	0.8972	0.9420
		G.E.B	5	14	0	1	0	0.7607	0.7778	0.8318
		Healthy	0	0	50	0	0	1.0000	1.0000	1.0000
		G.L.B	0	0	0	25	0	0.7540	0.7692	0.9537
		S.L.B	3	0	0	13	29	0.7621	0.7838	0.8028
✓	ST-R2	S.E.B	50	0	0	0	1	0.9111	0.9346	0.9687
		G.E.B	6	14	0	0	0	0.8224	0.8235	0.8367
		Healthy	0	0	50	0	0	1.0000	1.0000	1.0000
		G.L.B	0	0	0	25	0	0.9776	0.9804	0.9970
		S.L.B	0	0	0	1	44	0.9709	0.9778	0.9854

**Table 11:** Detailed performance comparison of different fine-tuning strategies and MCTS on 5-class potato dataset using Qwen3-VL-8B as the base model. (Part 2/2).

MCTS	FT strategy	Confusion matrices						Performance evaluation		
		Actual↓/Predict →	S.E.B	G.E.B	Healthy	G.L.B	S.L.B	MCC	F1	GM
✗	ST-R3	S.E.B	48	1	0	0	2	0.8946	0.9231	0.9527
		G.E.B	4	16	0	0	0	0.8539	0.8649	0.8918
		Healthy	0	0	50	0	0	1.0000	1.0000	1.0000
		G.L.B	0	0	0	24	1	0.9540	0.9600	0.9768
		S.L.B	1	0	0	1	43	0.9280	0.9451	0.9674
✓	ST-R3	S.E.B	49	0	0	0	2	0.9336	0.9515	0.9696
		G.E.B	3	17	0	0	0	0.9140	0.9189	0.9220
		Healthy	0	0	50	0	0	1.0000	1.0000	1.0000
		G.L.B	0	0	0	24	1	0.9769	0.9796	0.9798
		S.L.B	0	0	0	0	45	0.9582	0.9677	0.9897
✗	ST-R4	S.E.B	47	1	0	0	3	0.8804	0.9126	0.9427
		G.E.B	5	14	0	0	1	0.7901	0.8000	0.8342
		Healthy	0	0	50	0	0	1.0000	1.0000	1.0000
		G.L.B	0	0	0	23	2	0.9300	0.9388	0.9563
		S.L.B	0	0	0	1	44	0.9042	0.9263	0.9683
✓	ST-R4	S.E.B	47	0	0	0	4	0.8804	0.9126	0.9427
		G.E.B	5	14	0	0	1	0.8224	0.8235	0.8367
		Healthy	0	0	50	0	0	1.0000	1.0000	1.0000
		G.L.B	0	0	0	24	1	0.9769	0.9796	0.9798
		S.L.B	0	0	0	0	45	0.9198	0.9375	0.9792

To validate the effectiveness of complex multimodal information guided by MCTS search paths, this study further compares the performance of direct visual fine-tuning (Visual FT), multimodal fine-tuning.

(Multimodal FT), and self-training methods (ST-R1, ST-R2) on the potato disease classification task. Specifically, visual fine-tuning utilizes only image information to fine-tune the model, while multimodal fine-tuning directly employs the simple multimodal information from a potato leaf disease dataset (Zhu et al., 2025) for supervised training. In contrast, the self-training methods iteratively optimize the model using the complex multimodal information generated by MCTS, which is essentially a fine-tuning process oriented by the reliable paths explored by MCTS.

As shown in **Table 9**, the baseline model Qwen3-VL-8B achieved an accuracy of 0.5235 on the 5-class task and 0.6596 on the 3-class task. When augmented with MCTS, its performance improved significantly, reaching 0.9110 and 0.9319 accuracy, respectively. This indicates that MCTS effectively enhances the model’s decision-making process by exploring more reliable paths during inference.

Furthermore, the self-training agents demonstrated progressively better performance with increasing stages. ST-R1 achieved 0.7643 accuracy on the 5-class task, and ST-R2 further improved to 0.8691. When combined with MCTS, both agents achieved improvements: ST-R2 + MCTS reached 0.9581 accuracy on the 5-class task and 0.9948 on the 3-class task, while ST-R3 + MCTS achieved 0.9686 and 0.9895, respectively. These results suggest that self-training helps the model generate higher-quality pseudo-labels, thereby continuously improving accuracy.

In addition, visual fine-tuning achieves an accuracy of 0.5968 on the 5-class task, while multimodal fine-tuning achieves 0.5916. When combined with MCTS, the accuracy improves to 0.6073 for visual fine-tuning and 0.6283 for multimodal fine-tuning. Although both methods show improvements over the baseline model Qwen3-VL-8B (0.5235), the gains are limited. In contrast, the self-training methods ST-R1 and ST-R2 achieve accuracies of 0.7643 and 0.8691, respectively, which are significantly higher than those of visual fine-tuning and simple multimodal information fine-tuning. This fully demonstrates that complex multimodal information based on MCTS paths can fine-tune the model more effectively.

**Tables 10**, **11** provide a more granular view of the 5-class classification performance. For example, Visual FT + MCTS achieves a GM of 0.8788 on G.E.B categories, Multimodal FT + MCTS achieves an MCC of 0.4917 and a GM of 0.7706 on S.E.B categories and ST-R3 + MCTS achieves perfect recognition on the Healthy category (MCC = 1.0000). Similarly, ST-R4 + MCTS also attains a perfect score on the Healthy category and achieves an MCC of 0.9198 on the S.L.B category. In contrast, the baseline model without MCTS performs poorly on G.L.B and S.L.B, often misclassifying them as early blight or healthy, as reflected by its low MCC scores (e.g., 0.0000 for S.L.B). Visual fine-tuning completely fails to recognize G.L.B and S.L.B, with zero MCC scores for both categories, as a large number of late blight samples are misclassified as early blight. Multimodal fine-tuning also exhibits an MCC of zero for the S.L.B category and shows weak recognition capability for G.L.B. In comparison, the self-training methods ST-R1 through ST-R4 achieve non-zero MCC scores across all categories. Notably, ST-R4 achieves perfect recognition on the Healthy category (MCC = 1.0000) and attains MCC scores of 0.8804 and 0.9042 on S.E.B and S.L.B, respectively, demonstrating stronger robustness and balance.

Overall, the combination of self-training and MCTS consistently outperforms the baseline and the individual components, demonstrating strong potential for high-accuracy disease diagnosis in agricultural applications. Meanwhile, it also validates that complex multimodal information guided by MCTS paths has significant advantages over unimodal and simple multimodal information.

### 3.6 Evaluating the efficacy of MCTS in potato disease

To further validate the effectiveness of MCTS in enhancing the performance of vision-language models on the crop disease diagnosis task, we conducted comparative experiments using five representative baseline models: Qwen3-VL-8B, a vision-only fine-tuned model (Visual FT), a multimodal fine-tuned model (Multimodal FT), and four strong baselines, ST-R1, ST-R2, ST-R3 and ST-R4. All models were evaluated on a complete potato disease image dataset comprising five categories. The experimental results, presented in **Figure 8**, demonstrate that the introduction of MCTS leads to significant accuracy improvements across all models: Qwen3-VL-8B increased from 0.5235 to 0.9110, Visual FT from 0.5968 to 0.6073, Multimodal FT from 0.5916 to 0.6283, ST-R1 from 0.7643 to 0.9058, ST-R2 from 0.8691 to 0.9581, ST-R3 from 0.9476 to 0.9686.

**Figure 8:**
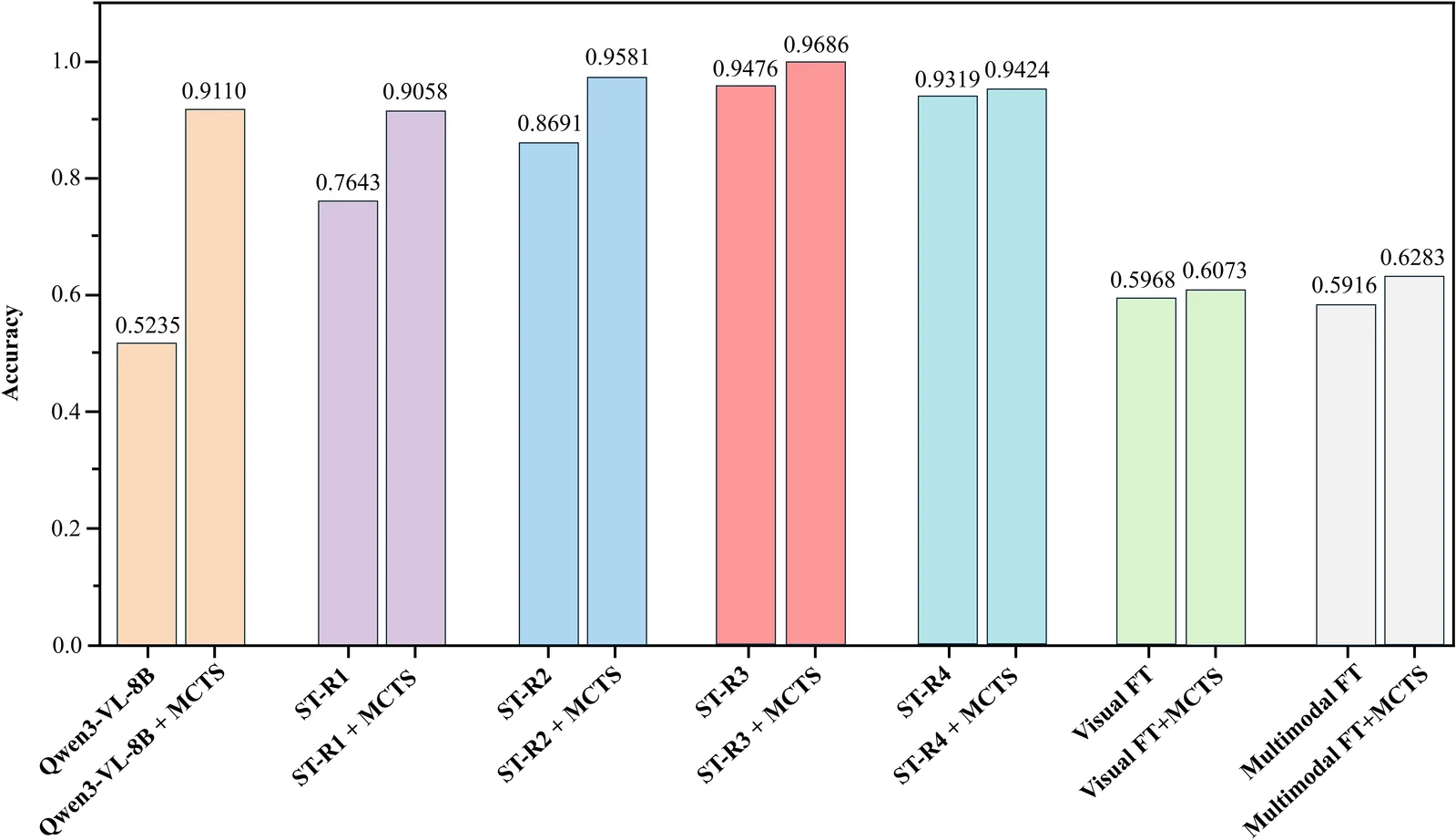
Performance comparison of different models with and without MCTS in 5-class potato disease dataset. (ST-Rn is a self-training classification agent (Qwen3-VL-8B) at stage n. Visual FT and Multimodal FT are fine-tuning methods based on Qwen3-VL-8B.).

These results strongly support the universality and effectiveness of MCTS across different vision-language models. Notably, the largest performance gain was observed for the base model Qwen3-VL-8B (+0.3875), indicating that MCTS can effectively compensate for the limitations of base models in visual feature extraction and reasoning capabilities by guiding them to focus on critical symptom features and generate coherent observation trajectories, thereby substantially enhancing diagnostic accuracy. For the fine-tuned strong baselines, such as ST-R3 and ST-R4, although their original performance was already high, MCTS still delivered significant gains of 0.021 and 0.0105, respectively. This suggests that, as a general-purpose reasoning enhancement module, MCTS can further exploit discriminative information by exploring more optimal sequences of observation actions, thereby pushing beyond the performance ceiling of fine-tuned models. In particular, ST-R3 combined with MCTS achieved an accuracy of 0.9686, approaching the saturation level of the task. Furthermore, a comparison between Visual FT and Multimodal FT reveals that the multimodal fine-tuned model attained a higher absolute performance when integrated with MCTS (0.6283), indicating that the synergy between multimodal information fusion and MCTS yields superior diagnostic outcomes. In summary, MCTS, as a plug-and-play reasoning optimization framework, effectively enhances the performance of various vision-language models on the crop disease diagnosis task, demonstrating strong generalizability and practical value.

### 3.7 Generalized application of our model for tomato disease classification

To further validate the generalization performance of the self-training fine-tuning strategy guided by MCTS paths, this study applies the proposed method to the tomato disease classification task. **Tables 12** and **13** summarizes the detailed evaluation metrics of different methods on five tomato disease categories (B.S; E.B; Healthy; L.B; Y.L.C.V).

**Table 12:** Performance comparison of different fine-tuning strategies and MCTS on tomato disease datasets using Qwen3-VL-8B as the base model. (Part 1/2).

MCTS	FT Strategy	Diseases	MCC	F1	GM	Pre	Recall	ACC	Overall_ACC
✗	✗	B.S	0.3333	0.3175	0.4438	0.7692	0.2000	0.2000	0.5280
		E.B	0.4491	0.5600	0.7723	0.4200	0.8400	0.8400	
		Healthy	0.6042	0.6714	0.8590	0.5222	0.9400	0.9400	
		L.B	0.0000	0.0000	0.0000	0.0000	0.0000	0.0000	
		Y.L.C.V	0.6040	0.6804	0.7835	0.7021	0.6600	0.6600	
✓	✗	B.S	0.4491	0.3871	0.4899	1.0000	0.2400	0.2400	0.5800
		E.B	0.7149	0.7719	0.8899	0.6875	0.8800	0.8800	
		Healthy	0.6913	0.7460	0.8965	0.6184	0.9400	0.9400	
		L.B	0.0000	0.0000	0.0000	0.0000	0.0000	0.0000	
		Y.L.C.V	0.4588	0.5676	0.7777	0.4286	0.8400	0.8400	
✗	ST-R1	B.S	0.7972	0.8367	0.8896	0.8542	0.8200	0.8200	0.7320
		E.B	0.6484	0.7059	0.8818	0.5581	0.9600	0.9600	
		Healthy	0.8614	0.8850	0.9670	0.7937	1.0000	1.0000	
		L.B	0.0000	0.0000	0.0000	0.0000	0.0000	0.0000	
		Y.L.C.V	0.8172	0.8544	0.9167	0.8302	0.8800	0.8800	
✓	ST-R1	B.S	0.8244	0.8571	0.9449	0.7742	0.9600	0.9600	0.7960
		E.B	0.8932	0.9143	0.9625	0.8727	0.9600	0.9600	
		Healthy	0.9306	0.9434	0.9849	0.8929	1.0000	1.0000	
		L.B	0.3136	0.2143	0.3464	1.0000	0.1200	0.1200	
		Y.L.C.V	0.7274	0.7769	0.9095	0.6620	0.9400	0.9400	
✗	ST-R2	B.S	0.8724	0.8972	0.9575	0.8421	0.9600	0.9600	0.8800
		E.B	0.8244	0.8571	0.9449	0.7742	0.9600	0.9600	
		Healthy	0.9415	0.9524	0.9874	0.9091	1.0000	1.0000	
		L.B	0.6667	0.6667	0.7071	1.0000	0.5000	0.5000	
		Y.L.C.V	0.9629	0.9703	0.9850	0.9608	0.9800	0.9800	
✓	ST-R2	B.S	0.8724	0.8972	0.9575	0.8421	0.9600	0.9600	0.9360
		E.B	0.9749	0.9796	0.9798	1.0000	0.9600	0.9600	
		Healthy	0.9757	0.9804	0.9950	0.9615	1.0000	1.0000	
		L.B	0.8598	0.8764	0.8832	1.0000	0.7800	0.7800	
		Y.L.C.V	0.9283	0.9423	0.9775	0.9074	0.9800	0.9800	
✗	ST-R3	B.S	0.8429	0.8727	0.9499	0.8000	0.9600	0.9600	0.8920
		E.B	0.8189	0.8409	0.8581	0.9737	0.7400	0.7400	
		Healthy	0.9640	0.9709	0.9925	0.9434	1.0000	1.0000	
		L.B	0.7464	0.7959	0.8631	0.8125	0.7800	0.7800	
		Y.L.C.V	0.9629	0.9703	0.9850	0.9608	0.9800	0.9800	
✓	ST-R3	B.S	0.8932	0.9143	0.9625	0.8727	0.9600	0.9600	0.9240
		E.B	0.8189	0.8409	0.8581	0.9737	0.7400	0.7400	
		Healthy	1.0000	1.0000	1.0000	1.0000	1.0000	1.0000	
		L.B	0.8485	0.8785	0.9450	0.8246	0.9400	0.9400	
		Y.L.C.V	0.9750	0.9800	0.9875	0.9800	0.9800	0.9800	

**Table 13:** Performance comparison of different fine-tuning strategies and MCTS on tomato disease datasets using Qwen3-VL-8B as the base model. (Part 2/2).

MCTS	FT Strategy	Diseases	MCC	F1	GM	Pre	Recall	ACC	Overall_ACC
✗	ST-R4	B.S	0.9173	0.9333	0.9750	0.8909	0.9800	0.9800	0.9320
		E.B	0.8456	0.8667	0.8810	0.9750	0.7800	0.7800	
		Healthy	0.9877	0.9901	0.9975	0.9804	1.0000	1.0000	
		L.B	0.8554	0.8846	0.9398	0.8519	0.9200	0.9200	
		Y.L.C.V	0.9750	0.9800	0.9875	0.9800	0.9800	0.9800	
✓	ST-R4	B.S	0.9629	0.9703	0.9850	0.9608	0.9800	0.9800	0.9480
		E.B	0.8598	0.8764	0.8832	1.0000	0.7800	0.7800	
		Healthy	1.0000	1.0000	1.0000	1.0000	1.0000	1.0000	
		L.B	0.8801	0.9009	0.9721	0.8197	1.0000	1.0000	
		Y.L.C.V	0.9875	0.9899	0.9899	1.0000	0.9800	0.9800	

In terms of overall accuracy, the baseline model Qwen3-VL-8B achieves only 0.5280, and completely fails to recognize L.B, with MCC, F1, Recall, and GM scores all being zero. After incorporating MCTS, the overall accuracy improves to 0.5800; however, the recall for L.B remains zero, indicating that relying solely on MCTS inference is insufficient to address the identification of challenging samples.

Based on the above findings, the self-training methods demonstrate significant performance improvements. ST-R1 achieves an overall accuracy of 0.7320, which further increases to 0.8800 for ST-R2, 0.8920 for ST-R3, and 0.9320 for ST-R4, reflecting the favorable convergence property of the self-training process. When combined with MCTS, the performance improves to 0.7960 for ST-R1+MCTS, 0.9360 for ST-R2 + MCTS, 0.9240 for ST-R3 + MCTS, and 0.9480 for ST-R4 + MCTS.

These results demonstrate that the self-training fine-tuning strategy based on MCTS paths not only performs excellently in potato disease classification but also exhibits strong generalization capability MCTS on tomato using Qwen3-VL-8B as the base model. (Part 1/2) on tomato, a different Solanaceae crop. The proposed method can be effectively transferred to disease recognition tasks across different crops, with particularly notable improvements in identifying challenging samples such as L.B, thereby fully validating the robustness and generalizability of the model.

### 3.8 Generalized application of our model for grape disease classification

To further validate the generalization ability of the proposed framework in plant disease recognition tasks, we selected grape, a plant species phylogenetically distant from previously tested Solanaceae crops (tomato and potato), as a new experimental subject. Grape diseases exhibit significant differences from Solanaceae crops in terms of pathological characteristics, infection sites, and visual appearances, providing a more rigorous test of the model’s cross-species adaptability. Experiments were conducted on a grape disease dataset comprising four categories: B.R, L.B, Healthy, and Esca.

**Table 14** show that the baseline model Qwen3-VL-8B achieves an overall accuracy of only 0.4688, with complete failure in recognizing B.R and Esca (MCC and F1 scores of 0), indicating its significant limitations in grape disease identification tasks. In contrast, as self-training progresses, the models exhibit excellent generalization performance: ST-R2 and ST-R3 achieve overall accuracies of 0.9843 and 0.9896, respectively. When further combined with MCTS inference, ST-R1 + MCTS achieves an overall accuracy of 0.5469, showing moderate improvement over the baseline. Notably, ST-R3 + MCTS achieved an overall accuracy of 0.9948, with scores of 1.0000 on L.B and Healthy across all metrics, and an MCC of 0.9867, an F1 of 0.9901, and a recall of 1.0000 on Esca.

**Table 14:** Performance comparison of different fine-tuning strategies and MCTS on grape disease datasets using Qwen3-VL-8B as the base model.

MCTS	FT Strategy	Diseases	MCC	F1	GM	Pre	Recall	ACC	Overall_ACC
✗	✗	B.R	0.0000	0.0000	0.0000	0.0000	0.0000	0.0000	0.4688
		L.B	0.2903	0.4925	0.5384	0.3289	0.9800	0.9800	
		Healthy	0.9548	0.9647	0.9814	0.9535	0.9762	0.9762	
		Esca	0.0000	0.0000	0.0000	0.0000	0.0000	0.0000	
✓	✗	B.R	0.0000	0.0000	0.0000	0.0000	0.0000	0.0000	0.4740
		L.B	0.3092	0.4975	0.5373	0.3311	1.0000	1.0000	
		Healthy	0.9847	0.9880	0.9880	1.0000	0.9762	0.9762	
		Esca	0.0000	0.0000	0.0000	0.0000	0.0000	0.0000	
✗	ST-R1	B.R	0.0000	0.0000	0.0000	0.0000	0.0000	0.0000	0.4792
		L.B	0.3188	0.5025	0.5503	0.3356	1.0000	1.0000	
		Healthy	0.9850	0.9882	0.9967	0.9767	1.0000	1.0000	
		Esca	0.0000	0.0000	0.0000	0.0000	0.0000	0.0000	
✓	ST-R1	B.R	0.3133	0.2951	0.4213	0.8182	0.1800	0.1800	0.5469
		L.B	0.3952	0.5464	0.6446	0.3759	1.0000	1.0000	
		Healthy	0.9705	0.9767	0.9933	0.9545	1.0000	1.0000	
		Esca	0.2458	0.1481	0.2828	1.0000	0.0800	0.0800	
✗	ST-R2	B.R	0.9595	0.9691	0.9695	1.0000	0.9400	0.9400	0.9843
		L.B	0.9867	0.9901	0.9965	0.9804	1.0000	1.0000	
		Healthy	1.0000	1.0000	1.0000	1.0000	1.0000	1.0000	
		Esca	0.9737	0.9804	0.9929	0.9615	1.0000	1.0000	
✓	ST-R2	B.R	0.9730	0.9796	0.9798	1.0000	0.9600	0.9600	0.9896
		L.B	1.0000	1.0000	1.0000	1.0000	1.0000	1.0000	
		Healthy	1.0000	1.0000	1.0000	1.0000	1.0000	1.0000	
		Esca	0.9737	0.9804	0.9929	0.9615	1.0000	1.0000	
✗	ST-R3	B.R	0.9730	0.9800	0.9865	0.9800	0.9800	0.9800	0.9896
		L.B	0.9865	0.9899	0.9899	1.0000	0.9800	0.9800	
		Healthy	1.0000	1.0000	1.0000	1.0000	1.0000	1.0000	
		Esca	0.9867	0.9901	0.9965	0.9804	1.0000	1.0000	
✓	ST-R3	B.R	0.9865	0.9899	0.9899	1.0000	0.9800	0.9800	0.9948
		L.B	1.0000	1.0000	1.0000	1.0000	1.0000	1.0000	
		Healthy	1.0000	1.0000	1.0000	1.0000	1.0000	1.0000	
		Esca	0.9867	0.9901	0.9965	0.9804	1.0000	1.0000	
✗	ST-R4	B.R	0.9079	0.9320	0.9624	0.9057	0.9600	0.9600	0.9583
		L.B	0.9189	0.9362	0.9381	1.0000	0.8800	0.8800	
		Healthy	1.0000	1.0000	1.0000	1.0000	1.0000	1.0000	
		Esca	0.9610	0.9709	0.9894	0.9434	1.0000	1.0000	
✓	ST-R4	B.R	0.9222	0.9423	0.9724	0.9074	0.9800	0.9800	0.9635
		L.B	0.9189	0.9362	0.9381	1.0000	0.8800	0.8800	
		Healthy	1.0000	1.0000	1.0000	1.0000	1.0000	1.0000	
		Esca	0.9737	0.9804	0.9929	0.9615	1.0000	1.0000	

Furthermore, ST-R4 + MCTS achieves an overall accuracy of 0.9635, with perfect recognition on Healthy and strong performance on Esca and B.R. These results demonstrate that our framework not only performs well on Solanaceae crops but also generalizes stably to the distantly related Vitaceae family, exhibiting robust cross-species generalization capability.

### 3.9 Impact of Self-training strategy in the proposed framework

The self-training convergence behavior of our proposed framework is comprehensively evaluated across multiple datasets and classification tasks, as illustrated in **Figure 9**.

**Figure 9:**
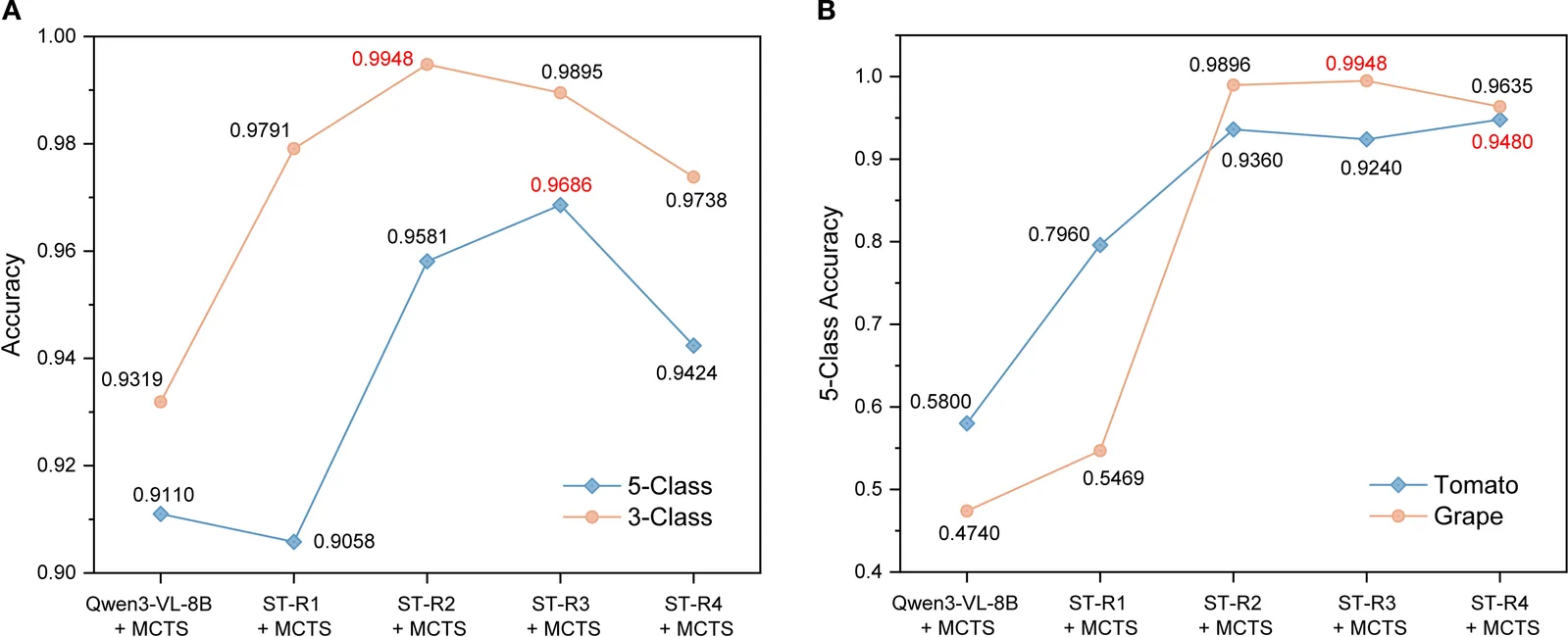
Convergence performance of the framework on different datasets and classification tasks. **(A)** Accuracy convergence on potato dataset (5-class and 3-class tasks). **(B)** Overall accuracy convergence on tomato and grape datasets.

On the potato dataset, as shown in **Figure 9A**, the proposed framework exhibits rapid convergence and high stability for both 5-class and 3-class classification tasks. In the initial iteration, the 5-class task achieves an accuracy of 0.9110, while the 3-class task reaches 0.9319. As the iterative self-training process progresses, different baseline models combined with MCTS all achieve significant improvements. Among them, ST-R2 + MCTS achieves the highest accuracy of 0.9948 on the 3-class task, and ST-R3 + MCTS achieves the highest accuracy of 0.9686 on the 5-class task. In the final iteration, the 3-class and 5-class tasks maintain accuracies of 0.9738 and 0.9424, respectively, demonstrating the framework’s robust convergence ability to maintain relatively high performance even after multiple training rounds.

As illustrated in **Figure 9B**, on the tomato dataset, the framework demonstrates effective convergence capability. Among the models, ST-R4 + MCTS achieves the best performance with an accuracy of 0.9480, followed by ST-R2 + MCTS with an accuracy of 0.9360, while ST-R1 + MCTS, ST-R3 + MCTS and Qwen3-VL-8B + MCTS achieve 0.7960, 0.9240 and 0.5800, respectively. On the Grape dataset, the ST-R3 + MCTS framework achieved the highest accuracy (0.9948), followed by ST-R2 + MCTS (0.9896) and ST-R4 + MCTS (0.9635). These results collectively confirm that the proposed self-training framework can effectively converge to high-performance states across different crop datasets, validating its strong generalization and convergence capabilities. Furthermore, we observe that the convergence performance varies significantly across different crops, which may be attributed to differences in the visual complexity and discriminability of disease symptoms, thereby affecting the learning difficulty and final diagnostic accuracy of the models.

However, self-training performance does not improve monotonically with increasing iteration rounds. A detailed analysis of this non-monotonic behavior is provided in Section 4.1. Given this non-monotonic characteristic, it is essential to determine the optimal stopping stage during training to ensure reliable deployment. In practice, where test labels may be unavailable, our framework addresses this by using a validation set during the training phase to evaluate model performance at each stage and select the best-performing stage as the final deployment model.

## 4 Discussion

### 4.1 Interpretation of classification performance

As shown in **Table 7**, our framework achieves 0.9965 on the 3-class potato task, surpassing existing task-specific models, but reaches only 0.9651 on the 5-class task, still below the state-of-the-art level of 0.9843.

This performance gap can be explained from two perspectives. First, during testing, the optimal path is selected based on the model’s confidence in the predicted category rather than the ground-truth label. Although the classifier agent undergoes multiple rounds of self-training and achieves improved overall capability, it may still exhibit overconfidence in certain incorrect predictions (Xu et al., 2024). This issue is particularly pronounced in the fine-grained 5-class task, where the visual differences between general and severe grades are subtle and easily confused. In contrast, the 3-class task benefits from substantially different visual patterns across disease types, allowing the model to establish a more stable decision boundary with lower overconfidence risk.

Second, generating reasoning chains inherently requires the executor agent to convert visual evidence into textual descriptions. Even though our framework adopts a region-specific description strategy rather than global description, this vision-to-text conversion inevitably loses some fine-grained visual information (Kodathala and Vunnam, 2025), leading to a certain accuracy loss.

In summary, in practical application scenarios such as grassroots agricultural extension and expert review, the transparent decision-making process provided by our framework offers clear practical value. Although enhancing interpretability may compromise classification accuracy to some extent, the framework still effectively assists diagnosticians in extracting key visual features and identifying potential errors, while maintaining reliable classification performance. In doing so, it strikes a beneficial balance between accuracy and interpretability, ultimately improving overall diagnostic efficiency and reliability.

### 4.2 Performance degradation in Self-Training and weakened effectiveness of MCTS

As shown in **Table 8**, the self-training process does not exhibit strictly monotonic performance improvements across all datasets. For instance, on the 5-class potato task, performance improves from ST-R1 (0.9058) to ST-R2 (0.9602) and ST-R3 (0.9651), but drops back to 0.9459 at ST-R4. This degradation can be attributed to the class imbalance in the training set at the ST-R4 stage.

Specifically, the class distribution of the ST-R4 training set is shown in **Figure 10**. The S.L.B class accounts for a significantly larger proportion (254 samples), while the healthy (18) and G.E.B (26) classes are severely underrepresented. This imbalanced distribution causes the model trained at ST-R4 to further enhance its discriminative ability for S.L.B, but at the cost of performance on other classes—particularly G.E.B. With only 26 training samples, the G.E.B class cannot be adequately learned, resulting in a notable performance drop at ST-R4. In contrast, the healthy class remains easily identifiable even with very few training samples, due to its distinct visual boundaries from other disease types. Therefore, the class distribution discrepancy directly leads to the performance degradation observed at ST-R4.

**Figure 10:**
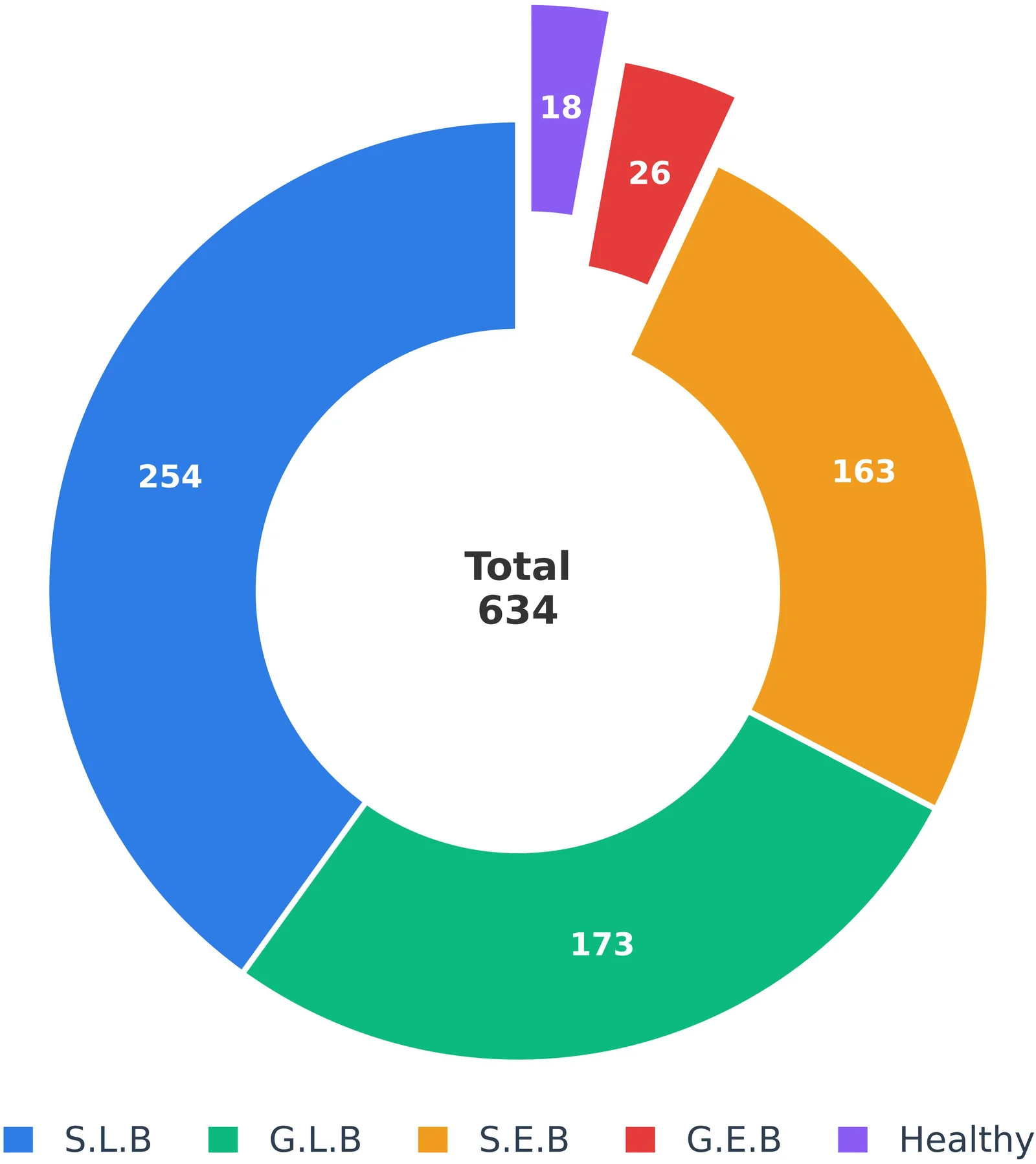
Class distribution of the ST-R4 training set. The dataset contains 18 Healthy, 26 G.E.B, 173 G.L.B, 254 S.L.B, and 163 S.E.B samples.

Furthermore, once the classifier’s discriminative capability approaches saturation, its ability to distinguish across different paths reaches its upper bound. Regardless of which observation paths MCTS discovers, the confidence scores produced by the classifier tend to converge with minimal differences across paths, thereby weakening the effectiveness of MCTS’s confidence-based path selection mechanism.

### 4.3 Limitations and risks

All experiments in this study are conducted on the PlantVillage dataset, which consists of leaf images captured under controlled laboratory conditions. While PlantVillage is a widely adopted benchmark in plant disease recognition, it does not fully capture the complexity of real-world agricultural environments.

In terms of interpretability, our framework is inherently capable of generating reasoning chains, thereby enhancing decision-making transparency. However, due to the lack of standardized quantitative metrics for evaluating interpretability, which remains an open challenge in the field, we only provide qualitative demonstrations of reasoning chains through representative case studies without offering quantitative assessments of their quality.

Furthermore, reasoning chains are generated by the executor agent based on MLLM-based visual perception, and their descriptions may deviate from the actual visual evidence or even produce hallucinations. Therefore, in practical applications, particularly in high-stakes decision-making scenarios, we recommend adopting larger-scale models to improve description accuracy, or incorporating human expert review mechanisms to ensure diagnostic reliability.

Additionally, a systematic sensitivity analysis of the MCTS reward hyperparameters (shown in **Table 3**) during the training phase was not conducted in this study. Although the current empirical settings have proven effective in guiding the search, a comprehensive sensitivity analysis could further reveal the impact of reward values on framework performance and provide stronger justification for the reward function design.

## 5 Conclusions

This study proposes an intelligent classification framework for crop disease recognition that integrates MCTS, multimodal information, multi-agent collaboration, and a self-training mechanism, thereby simultaneously addressing the insufficient classification accuracy of MLLMs and the lack of interpretability of task-specific models. The framework leverages MCTS for interpretable reasoning and employs multiagent collaboration to generate region-specific text descriptions, progressively enhancing multimodal information. Combined with a self-training strategy for iterative performance improvement, the framework improves classification accuracy while generating traceable reasoning chains. Experimental results show that on the potato dataset, the framework achieves 0.9965 and 0.9651 accuracy on the 3-class and 5-class tasks, respectively. On the tomato and grape datasets, it achieves 0.9467 and 0.9948 accuracy, respectively, demonstrating excellent cross-crop generalization capability. Our approach offers a viable solution for high-precision, interpretable disease recognition in complex agricultural scenarios.

## 6 Future work

Despite the promising results, several limitations of the current framework remain to be addressed in future work. First, we plan to validate the framework on field-captured images to assess its generalization beyond the controlled PlantVillage dataset. Second, we intend to explore domain-specific quantitative metrics for evaluating interpretability in plant pathology, as standardized measures are currently lacking. Third, we plan to extend the framework from classification to detection and segmentation tasks by introducing external tool support and constructing a detection agent based on MCTS and multimodal large models. This agent will enable multi-perspective evidence collection and multi-scale information fusion, thereby improving reasoning depth and decision-making robustness in complex environments.

## Data Availability

The raw data supporting the conclusions of this article will be made available by the authors, without undue reservation.
